# Integrated network pharmacology and experimental validation to explore the mechanisms underlying naringenin treatment of chronic wounds

**DOI:** 10.1038/s41598-022-26043-y

**Published:** 2023-01-04

**Authors:** Rui Sun, Chunyan Liu, Jian Liu, Siyuan Yin, Ru Song, Jiaxu Ma, Guoqi Cao, Yongpan Lu, Guang Zhang, Zhenjie Wu, Aoyu Chen, Yibing Wang

**Affiliations:** 1 Department of Plastic Surgery, Shandong Provincial Qianfoshan Hospital, Shandong University, Jinan, Shandong 250012 People’s Republic of China; 2 Department of Plastic Surgery, The First Affiliated Hospital of Shandong First Medical University & Shandong Provincial Qianfoshan Hospital, Jinan, Shandong 250014 People’s Republic of China; 3 The First Clinical Medical College, Shandong University of Traditional Chinese Medicine, Jinan, Shandong 250014 People’s Republic of China; 4 Jinan Clinical Research Center for Tissue Engineering Skin Regeneration and Wound Repair, Jinan, Shandong 250014 People’s Republic of China

**Keywords:** Computational biology and bioinformatics, Plant sciences, Diseases

## Abstract

Naringenin is a citrus flavonoid with various biological functions and a potential therapeutic agent for skin diseases, such as UV radiation and atopic dermatitis. The present study investigates the therapeutic effect and pharmacological mechanism of naringenin on chronic wounds. Using network pharmacology, we identified 163 potential targets and 12 key targets of naringenin. Oxidative stress was confirmed to be the main biological process modulated by naringenin. The transcription factor p65 (*RELA*), alpha serine/threonine-protein kinase (*AKT1*), mitogen-activated protein kinase 1 (*MAPK1*) and mitogen-activated protein kinase 3 (*MAPK3*) were identified as common targets of multiple pathways involved in treating chronic wounds. Molecular docking verified that these four targets stably bound naringenin. Naringenin promoted wound healing in mice in vivo by inhibiting wound inflammation. Furthermore, in vitro experiments showed that a low naringenin concentration did not significantly affect normal skin cell viability and cell apoptosis; a high naringenin concentration was cytotoxic and reduced cell survival by promoting apoptosis. Meanwhile, comprehensive network pharmacology, molecular docking and in vivo and in vitro experiments revealed that naringenin could treat chronic wounds by alleviating oxidative stress and reducing the inflammatory response. The underlying mechanism of naringenin in chronic wound therapy involved modulating the RELA, AKT1 and MAPK1/3 signalling pathways to inhibit ROS production and inflammatory cytokine expression.

## Introduction

Normal skin covers the surface of the human body, serves as a protective barrier against the invasion of pathogens, adapts to changes in the external environment, and plays vital roles in body homeostasis. Following trauma, burns, operation, and complications of many chronic diseases, such as diabetes and peripheral vascular diseases, the anatomic and functional integrity of skin can be damaged, and the sophisticated process of normal wound healing is activated^1^. Normal skin repair requires the epidermis, dermis, subcutaneous adipose tissue and resident immune cells to perfectly coordinate in sequential stages. These stages of haemostasis, inflammation, proliferation, and remodelling occur in a temporal sequence but show some overlap^2^. When the normal repair process is disturbed, it may lead to chronic wounds, such as diabetic foot, venous leg ulcers, and pressure sores^3^. Although a standardized, global consensus to define chronic wounds has not yet been achieved^4^, many researchers and clinicians regard a wound as a chronic wound when it has not healed within 1 month^5, 6^. Studies have indicated that the incidence of chronic wounds is increasing due to the increasing number of older adults and the presence of underlying chronic comorbidities^7^. Statistics show that the prevalence of chronic wounds worldwide is approximately 2.21 per 1000 people in a population^8^. In the United States, 3% of the population older than 65 years has open wounds^9^. However, the precise pathogenesis of chronic wounds remains unclear. Despite adequate wound management, chronic wounds remain intractable^10^. For these reasons, chronic skin wounds have gradually become global health problems imposing increasing psychological, physical, and economic burdens on millions of people worldwide and bring great challenges to healthcare systems^11, 12^. Studies have shown that wound management accounts for more than half of the community health nurse resources in European settings^13^. Moreover, 27% to 50% of hospital beds are occupied by patients requiring some form of wound management^14^. In the United Kingdom, the cost of a pressure ulcer increases with severity from $1600 to $18,000^15^. The growing body of knowledge on this issue and related mechanistic research have indicated that in addition to good wound care, many new medical devices and drugs (i.e., dressings and topical agents) will be proposed for chronic wound treatment^16, 17^. The use of negative pressure wound therapy (NPWT) has reduced edema and the risk of infection, promoted angiogenesis, enhanced granulation tissue formation and improved patient quality of life in the last decade^18^. The rapid development of cell and tissue bioengineering has provided a novel approach to wound treatment; for instance, PermaDerm and denovoSkin are on their way to the clinic^19, 20^. However, the experimental nature of the treatments and the high costs limit the use of artificial tissue^21^. Due to their low immunogenicity, versatility, robustness and scalability, inorganic nanoparticle hybrid materials are used as drugs and carriers for chronic wound treatment^22^. Although an increasing number of therapies for chronic wound healing are available, a completely effective treatment is unavailable^17^.

Many natural products with therapeutic potential have been applied in clinical practice and have attracted increasing interest in recent years^23^. Meanwhile, an increasing number of natural products combined with other dressings and agents for the treatment of chronic wounds have achieved satisfactory results^24, 25^. Naringenin (the chemical structure is shown in Fig. 2a) is a traditional Chinese component commonly found in citrus fruits and is a naturally occurring flavonoid^26^. It has a molecular weight of 272.26 P(C_15_H_12_O_5_) and exists predominantly in nature in two forms: glycosylated (naringin or naringenin-7-O-glucoside) and aglycosylated (naringenin)^27^. Naringenin possesses various pharmacological properties, such as anti-inflammatory^28^, antioxidant^29^, antifibrotic^30^, neuroprotective^31^, antibacterial^32^ and anticancer properties^33^. Naringenin can protect the liver by inhibiting oxidative stress and the transforming growth factor (TGF-β) pathway and preventing the transdifferentiation of hepatic stellate cells (HSCs) to inhibit liver fibrosis^34^. Naringenin induces cell cycle arrest in G and S phases in hepatocellular carcinoma cells by inhibiting cyclin^35^, and naringenin can reduce the metastasis and invasion of pancreatic cells by reducing vascular endothelial growth factor and downregulating the TGF-β pathway, showing vascular inhibitory effect^36^. Naringenin might have a therapeutic effect against COVID-19 by inhibiting the major COVID-19 protease (3CLpro) and reducing angiotensin converting enzyme receptor activity^28^. Unfortunately, it has a short half-life and is quickly converted to crystalline form^37^. Only 15% of ingested naringenin is absorbed by the human gastrointestinal tract, and its bioavailability is very low, which limits its practical use^38^. In skin disease, different technologies or methods are combined to enhance the clinical applications of naringenin because of its low affinity for water^39^. For instance, a naringenin-loaded microsponge gel controls naringenin release, which may become a promising drug for atopic dermatitis^40^. A naringenin-loaded microemulsion sericin gel has shown therapeutic efficacy against UVB-induced photoaging^41^. In addition, alginate hydrogels containing naringenin accelerate the healing of excisional wounds in rats^42^. Based on the findings described above, we speculate that naringenin likely also has the potential to treat chronic wounds^43^. However, the underlying molecular mechanism of naringenin in chronic wound healing is still unknown.

**Figure 1:**
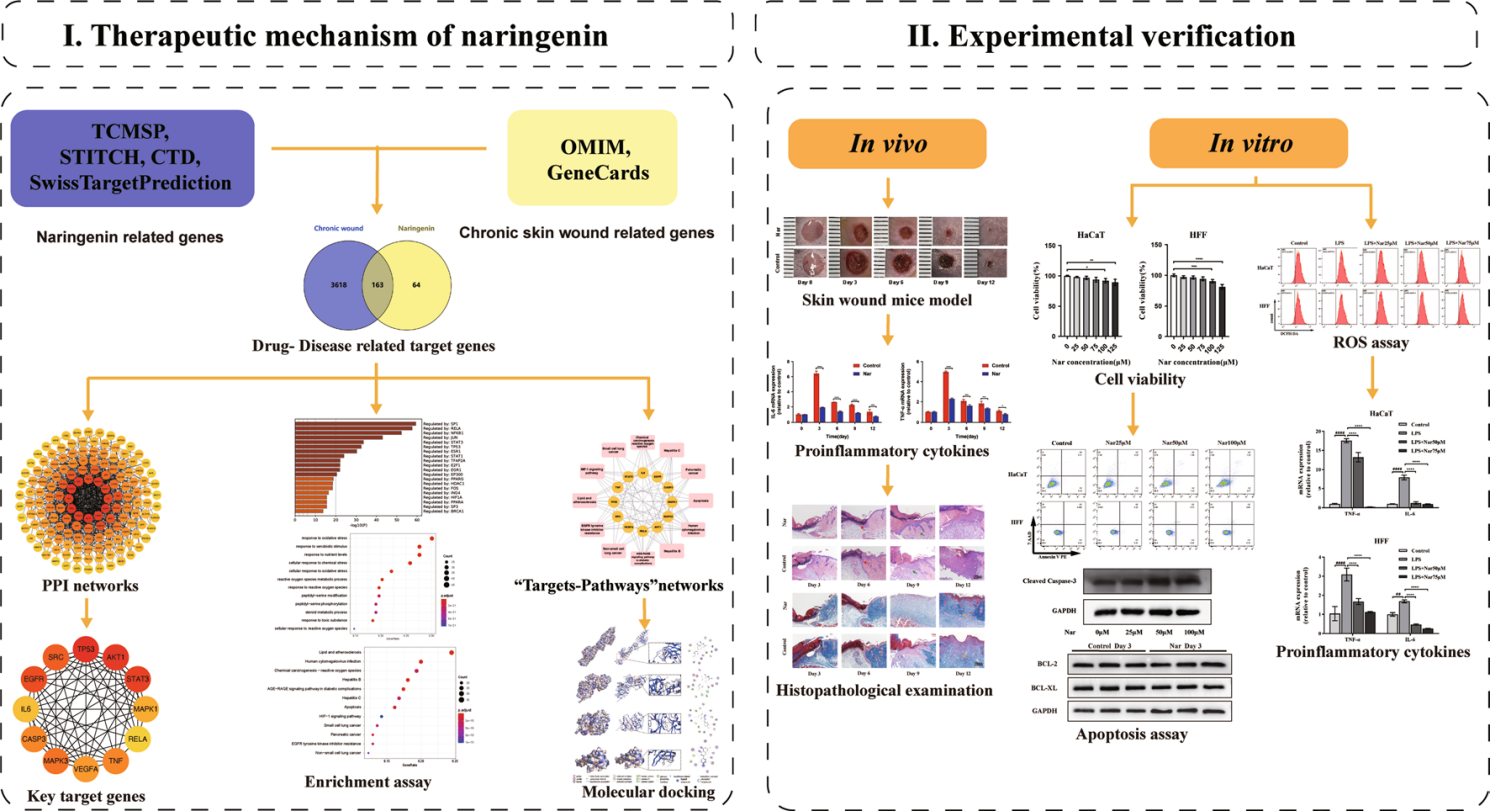
Workflow of the study.

Network pharmacology, a novel research tool, changes the traditional “one disease, one target, one drug” mode, and adopts a “multicompound, multitarget and multipathway” concept^44^. Network pharmacology, which was proposed by Hopkins in 2007^45^, combines systems biology, bioinformatics, and pharmacological approaches to achieve precise and effective therapeutic interventions, eliminate the need for drug discovery, and accelerate clinical translation^46^. Molecular docking is a computer technique that simulates drug- target interactions based on structural design, and can be integrated with network pharmacology for component prediction and mechanism study^47^. Meanwhile, network pharmacology represents a new approach to identify the active components in traditional Chinese medicines, along with biological targets and related signalling pathways, for the treatment of certain diseases^48^. This technique has been proven to work in a variety of herbs in traditional medicine^49–51^. Hence, the present study aimed to evaluate the ability of naringenin to promote chronic wound healing. Network pharmacology analysis and molecular docking were used to explore the underlying molecular targets and signalling pathways. Then, the pharmacological effects of naringenin on both in vivo and in vitro systems were further examined. The results suggest that naringenin is a promising drug for chronic wound treatment (Fig. 1).

## Methods

### Inquiry and clustering of the structure and target genes of naringenin

The targets of naringenin were obtained based on chemical similarities, pharmacophore models and protein interactions by searching the Traditional Chinese Medicine Systems Pharmacology Database and Analysis Platform (TCMSP) database (https://old.tcmsp-e.com/index.php)^52^, STITCH database (http://stitch.embl.de/cgi/)^53^, Comparative Toxicogenomics Database (CTD) (http://ctdbase.org/)^54^ and SwissTargetPrediction Database (http://www.swisstargetprediction.ch/)^55^. The UniProt database (https://www.uniprot.org/)^56^ was used to standardize canonical gene names and obtain the possible target genes of naringenin.

### Screening of candidate naringenin targets in chronic skin wounds

Different genes associated with chronic wounds were collected from the GeneCards database (https://www.genecards.org/)^57^ and Online Mendelian Inheritance in Man (OMIM) database (https://omim.org/)^58^. Duplicate targets were removed, and overlapping component-related and disease-related proteins were identified based on Venny 2.1 (https://bioinfogp.cnb.csic.es/tools/venny/)^59^ intersections as potential targets of naringenin in chronic skin wounds.

### Construction of the protein–protein interaction (PPI) network

A PPI network map was constructed in which two or more proteins form a protein complex for coexpression, neighbourhood, gene fusion and cooccurrence. The names of the potential target genes were reassessed using the Search Tool for the Retrieval of Interacting Genes (STRING) 11.5 database (https://cn.string-db.org/)^60^. “*Homo sapiens*” was chosen, and a score > 0.7 was selected as indicating a high confidence protein interaction. Furthermore, the result was visualized using Cytoscape 3.8.2 software^61^, and the CytoHubba plugin was used to screen key target proteins by analysing the genes whose degree values were greater than their respective medians.

### Analysis of the gene list from the metascape database

The Metascape database (https://metascape.org/)^62^, a gene annotation and analysis resource database, was used to perform an MCODE enrichment analysis and transcription factor (TF) enrichment analysis by integrating the potential target genes of naringenin in chronic skin wounds.

### GO and KEGG pathway enrichment analyses

The R package “org.Hs.eg.db, version 3.14” was used to transform potential target gene names into their Entrez IDs. Then, Gene Ontology (GO) enrichment analysis and Kyoto Encyclopedia of Genes and Genomes (KEGG) pathway enrichment analysis were performed using the R packages “DOSE”, “clusterProfiler^54^” and “ggplot2”, for which the *P* value was set to < 0.05 for further analysis. The network of the target pathways was established using Cytoscape 3.8.2 software.

### Molecular docking between naringenin and key target proteins

**Figure 2:**
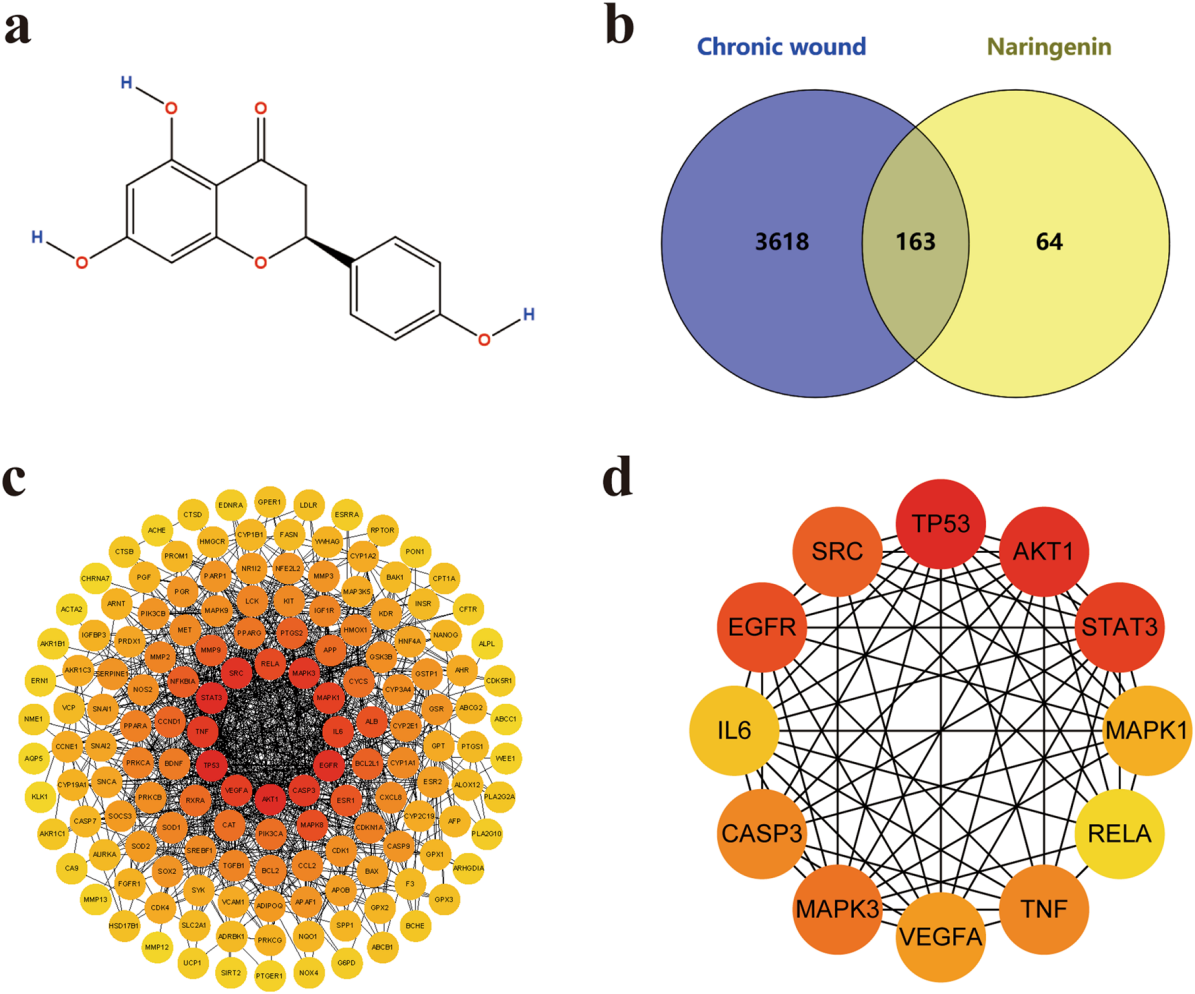
Targets of naringenin in treating chronic wounds. (**a**) 2D structure of naringenin. (**b**) Venn diagram depicting the 163 overlapping genes involved in the effects of naringenin on chronic wounds. (**c**) STRING analysis indicating the PPI network among the 163 overlapping targets of naringenin in chronic wound treatment. (**d**) Cytoscape analysis of the 12 key target networks involved in the effects of naringenin on chronic wounds.

**Figure 3:**
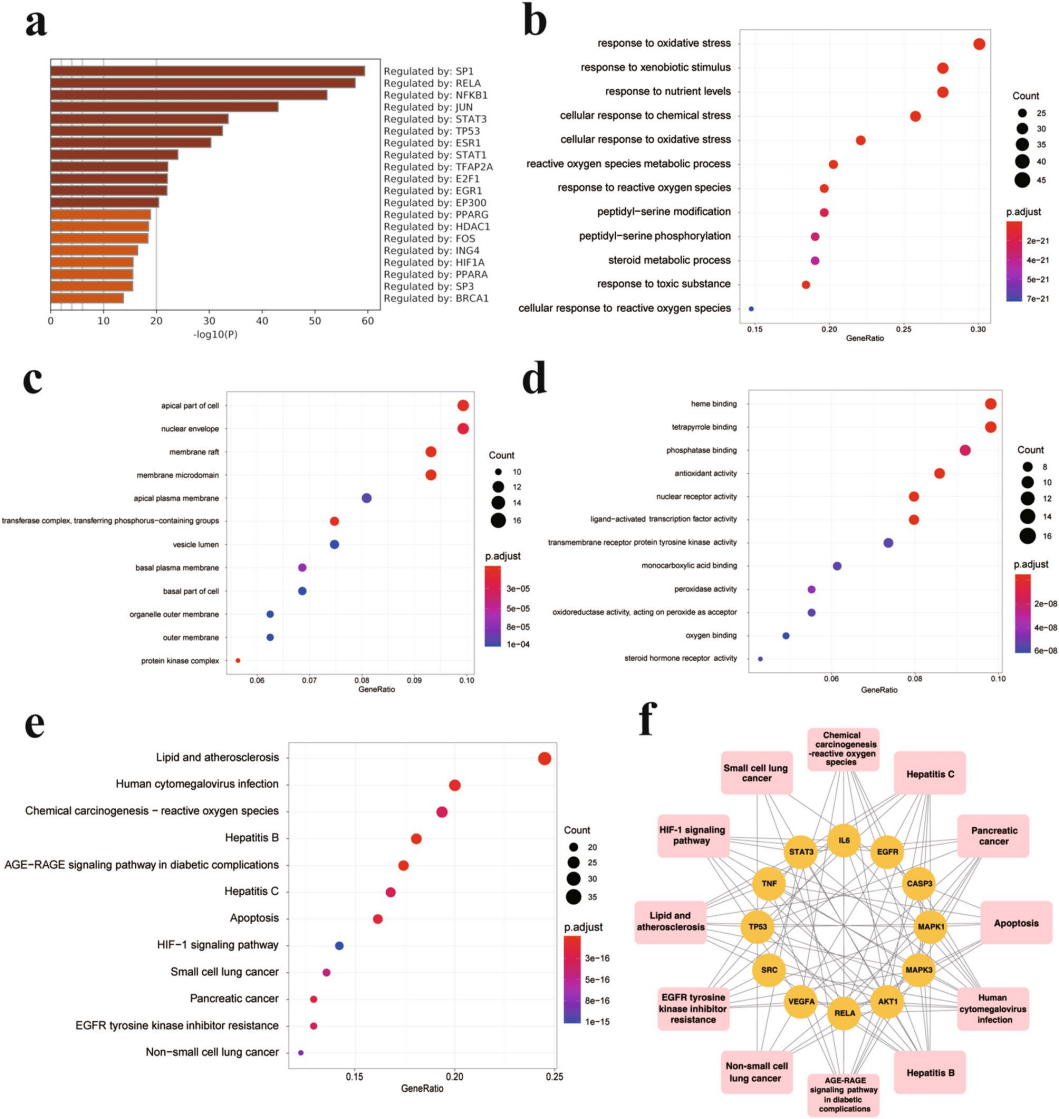
Enrichment analysis of naringenin in the treatment of chronic wounds. (**a**) TF enrichment analysis of 163 overlapping genes in the TRRUST database using Metascape. The bubble diagrams show the terms in the (**b**) biological process (BP), (**c**) cellular component (CC) and (**d**) molecular function (MF) categories in the GO analysis of 163 overlapping genes for the treatment of chronic wounds with naringenin. (**e**) The top 12 enriched KEGG pathways are highlighted in the bubble diagram. (**f**) The relations between the top 12 KEGG pathways and the key targets of naringenin.

The 2D (Fig. 2a) and 3D chemical structures of naringenin were obtained from PubChem (https://pubchem.ncbi.nlm.nih.gov/)^63^ in SDF file format. The structure files of key target proteins were retrieved from the Protein Data Bank database (https://www.rcsb.org/)^64^ in PDB format. The structure files of naringenin and target proteins were uploaded to CB-Dock2 (https://cadd.labshare.cn/cb-dock2/php/index.php)^65, 66^ to evaluate the binding capacity. CB-Dock2 was integrated with Open Babel and AutoDock Vina, which was used to analyse the interactions between key target proteins and naringenin, including determining binding pocket sites, molecular docking and the docking score^67^. Medicinal chemistry and the molecular operating environment (MOE) software^68^ was used to visualize the molecular docking results.

### Reagents

Naringenin (Cat No. HY-N0100) and Protease Inhibitor Cocktail (Cat No. HY-K0010) were purchased from MedChemExpress (NJ, USA). Cell Counting Kit-8 (CCK-8, CK04) was obtained from Dojindo (Japan). Reactive oxygen species (ROS) assay kits (S0033S), cell lysis buffers (P0013), and enhanced BCA protein assay kits (P0010S) were ordered from Beyotime Institute of Biotechnology (Shanghai, China). A SteadyPure Quick RNA Extraction Kit (Code No. AG21023) was purchased from Accurate Biotechnology Co., Ltd. (Hunan, China). A PE Annexin V Apoptosis Detection Kit I was obtained from BD Biosciences (San Jose, CA, USA). Lipopolysaccharide (LPS, L8880) was ordered from Solarbio^®^ Life Sciences (Beijing, China). Primary antibodies against AKT1 and phospho-AKT1 (Ser473) were supplied by Abcam. A primary antibody against caspase-3, BCL-2 and BCL-XL were supplied by Proteintech. Antibodies against NF-κB p65/RELA, phospho-NF-κB p65/RELA (Ser536), MAPK1/3, phospho-MAPK1/3 (Thr202/Thr204) and GAPDH were provided by Cell Signaling Technology (Beverly, MA, United States).

### Animal experiments and ethics statements

All animal experiments were approved by the Institutional Animal Care and Use Committee of Qianfoshan Hospital Affiliated with Shandong University (permit No. SYDWLS2021-002). All animal experiments were performed in accordance with the ARRIVE guidelines^69^ and in accordance with the National Institutes of Health Guide for the Care and Use of Laboratory Animals. Eighteen mice used were specific pathogen-free 7-week-old male C57BL/6J mice (weight, 20–25 g; Shandong University Laboratory Animal Centre, China). Before the experiment, all the animals were acclimated to the experimental conditions for 1 week.

All mice were anaesthetized with 3% isoflurane for more than 5 min using a small animal anesthesia system. The mice were placed in the prone position. The dorsal hair was shaved with an electric shaver and then removed with a depilatory cream. Within 1 min, gauze swabs were used to remove all cream and remaining fur. Then, the skin was disinfected with 75% ethanol, and rinsed with PBS. Two 5-mm circular full-thickness wounds were created on each side of the midline upper dorsal skin using ophthalmic scissors under sterile surgical conditions. Briefly, with one hand, a pair of ophthalmic toothed forceps was used to lift the skin on the left or right sides of the mouse's dorsal midline, and with the other hand, a pair of ophthalmic scissors was used to create a full-thickness wound and excise the circular piece of the skin. The procedure described was repeated to create a wound on the opposite side of the midline. The excised skins were saved as a baseline control (the day of surgery was designated as day 0). A total of 27.225 µg naringenin was dissolved in 1 ml DMSO to obtain 100 µM naringenin working solution. 10 µl of DMSO (control group) or 10 µl of 100 µM naringenin (naringenin group) was intracutaneously injected along a distance of 2 mm from the wound edge daily until the wound healed. The scab was carefully removed with sterile forceps before taking the photograph. A straight edge was placed next to the wound to correct for the distance between the camera and the animals. Photographs were obtained on the day of surgery and every 2 days after injury until the wounds completely healed. The wound bed sizes were quantified in pixels with ImageJ software (National Institutes of Health, Bethesda, MD, USA), and the residual defect areas were calculated using the equation: the residual defect areas = (actual wound area/original wound area) × 100%. After operation, each mouse was stored in a single cage. Mice were euthanized by excessive anesthesia at days 3, 6, 9 and 12, and wound margins were collected for analysis. The wound along with the 2 mm skin around the wound was completely excised with ophthalmic scissors. The cut skin samples were tested in subsequent operations.

### Histopathology and Masson trichrome staining

The skin tissue was fixed with 4% paraformaldehyde, dehydrated, and embedded in paraffin. Finally, a series of sections were collected with a 3 µm microtome. Haematoxylin and eosin (H&E) staining was used for general morphological observation, and Masson trichrome (MT) staining was used to evaluate granulation tissue. The slides were examined under a light microscope and micrographs were taken for analysis.

### Cell culture

The human epidermal keratinocyte cell line HaCaT obtained from GeneChem (Shanghai, China) was maintained in modified RPMI medium (1640, Gibco, USA) containing 10% foetal bovine serum (FBS). Normal human foreskin fibroblasts (HFFs) purchased from Cell Research (Shanghai, China) were passaged in high-glucose Dulbecco's modified Eagle's medium (DMEM, Gibco, USA) containing 15% FBS. Both cell types were cultured at 37 °C with 5% CO_2_.

### Cell viability analysis

Cell viability was evaluated using the Cell Counting Kit-8 (CCK-8) assay. A total of 27.225 mg naringenin was dissolved in 1 ml DMSO to obtain 100 mM naringenin concentrate. In subsequent cell experiments, the naringenin concentrate was diluted in medium to prepare suitable naringenin working solution. Cells in the logarithmic growth phase were seeded in 96-well plates (5 × 10^3^ cells per well) and cultured overnight at 37 °C with 5% CO_2_ in an incubator. The next day, the fresh medium was changed to different concentrations of naringenin (0, 25, 50, 75, 100, or 125 µM). After 24 h of culture, the medium was replaced with 110 µl of CCK-8 solution (10 µl/100 µl in fresh medium). After a 1 h incubation, the optical density (OD) value was measured at 450 nm, and the relative cell viability was calculated according to the manufacturer’s instructions.

### Apoptosis analysis

Cells were plated into 6-well plates for 24 h and treated with various concentrations of naringenin (0, 25, 50, or 100 μM) for 3 h. After stimulation, the cells were washed twice with cold PBS and then collected in a culture tube. Then, the cells were resuspended in 100 μl of 1X Binding Buffer containing 5 µl of PE-conjugated Annexin V and 5 µl of 7-AAD and incubated for 15 min at room temperature in the dark. Finally, 400 µl of 1× Binding Buffer were added to each culture tube to complete the staining. Ten thousand cells per tube were collected by cell Flex flow cytometer (Beckman Coulter, USA), and the percentage of apoptotic cells was determined.

### Determination of ROS generation

Changes in intracellular ROS levels were determined by measuring the oxidative conversion of the cell-permeable dye 2′,7′-DCF diacetate (DCFH-DA). A total of 2 × 10^5^ cells were seeded in 6-well plates and pretreated with various concentrations of naringenin for 3 h prior to exposure to LPS alone. After 12 h of LPS stimulation, the cells were washed with PBS and incubated with DCFH-DA at 37 °C for 30 min in the dark. After 3 washes with PBS, the cells were immediately detected using a CytoFLEX flow cytometer (Beckman Coulter, USA).

### Quantitative real-time polymerase chain reaction (qRT–PCR)

Total RNA was collected from mouse wound edge samples and cell samples. Total RNA was extracted using a SteadyPure Quick RNA Extraction Kit, and mRNA expression was quantified according to the manufacturer’s suggested program using an Applied Biosystems QuantStudio 3 (Thermo Scientific, MA, USA). The primers used were synthesized by BGI (Beijing, China). The mRNA expression levels were normalized to the expression of the endogenous control ACTB and analysed using the ΔΔCt method. Detailed information on the primer sequences is listed in Supplementary Table 1.

### Western blot analysis

Total protein was extracted from cells and skin tissues using cell lysis buffer containing protease inhibitor cocktail and a phosphatase inhibitor. An Enhanced BCA Protein Assay Kit was used to calculate the protein concentrations from the standard curves. Protein solutions were boiled in 5 × loading buffer at 98 °C for 10 min. The proteins (20 μg) were separated on sodium dodecyl sulfate–polyacrylamide gels and transferred to polyvinylidene fluoride membranes (Millipore, MA, USA), and the membranes were blocked with 5% skim milk for 2 h at room temperature. In order to improve the clarity and simplicity of the blots, the membrane was trimmed before hybridization with the primary antibody. Next, the membranes were incubated overnight at 4 °C with primary antibodies at a dilution of 1:1000. After washing with TBST, the membranes were incubated with horseradish peroxidase-conjugated secondary antibodies (1:5000) for 1 h at room temperature. Finally, the immunoreactive bands were imaged using a ChemiDoc™ Imaging System (Bio-Rad, CA, USA). ImageJ software was used for the densitometry analysis. The protein expression levels were normalized to the expression of the endogenous control GAPDH.

### Statistical analysis

Data were analysed using GraphPad Prism 8.0 software (GraphPad Software, Inc., USA). Data from a minimum of three experimental replicates are presented as the means ± standard deviations. Differences between two groups were evaluated using Student’s *t test*. Multiple-group comparisons were conducted using analysis of variance (ANOVA). *P < 0.05, **P < 0.01, ***P < 0.001, and ****P < 0.0001 were considered significant.

## Results

### Clustering of naringenin- and chronic skin wound-related target genes

Potential targets of naringenin were clustered using the TCMSP, STITCH, CTD and SwissTargetPrediction databases, and 227 targets were calibrated to standardize their names (after removing duplicates) using the UniProt database. The inclusion criteria for genes related to chronic skin wounds in the GeneCards database was a gene score > 1, and 3781 genes related to chronic skin wounds were collected from the GeneCards and OMIM databases. A Venn diagram of the two target sets was built to obtain 163 overlapping targets (Fig. 2b; Supplementary Table 2).

### PPI network map and the key targets

The 163 target genes were imported into the STRING database to obtain the PPI network map. Using a high confidence interaction (minimum required interaction score > 0.700), the PPI network of naringenin therapy for chronic skin wounds was generated, which consisted of 163 nodes, 951 edges, an average node degree of 11.7, an average local clustering coefficient of 0.473 and a PPI enrichment *P* value < 1.0e^−16^ (Fig. 2c). Sequentially, the mapped proteins were imported into Cytoscape software (version 3.8.2) to calculate the interaction network topological parameters for key proteins. The top 12 targets, namely, cellular tumor antigen p53 (TP53), RAC-alpha serine/threonine-protein kinase (AKT1), signal transducer and activator of transcription 3 (STAT3), epidermal growth factor receptor (EGFR), proto-oncogene protein tyrosine kinase Src (SRC), mitogen-activated protein kinase 3 (MAPK3), tumor necrosis factor (TNF), caspase-3 (CASP3), vascular endothelial growth factor A (VEGFA), mitogen-activated protein kinase 1 (MAPK1), interleukin-6 (IL-6), and the transcription factor p65 (RELA), may play important role in the network and were selected based on their higher degree scores (degree > 30) (Fig. 2d; Table 1).

**Table 1:** Top 12 high-degree genes in the network.

Rank	Name	Protein names	Score
1	TP53	Cellular tumor antigen p53	61
2	AKT1	RAC-alpha serine/threonine-protein kinase	58
3	STAT3	Signal transducer and activator of transcription 3	46
4	EGFR	Epidermal growth factor receptor	44
5	SRC	Proto-oncogene protein tyrosine kinase Src	43
6	MAPK3	Mitogen-activated protein kinase 3	39
7	TNF	Tumor necrosis factor	38
7	CASP3	Caspase-3	38
9	VEGFA	Vascular endothelial growth factor A	35
10	MAPK1	Mitogen-activated protein kinase 1	34
11	IL-6	Interleukin-6	32
12	RELA	Transcription factor p65	31

Next, the top 5 representative modules were extracted from the PPI network using MCODE, and the module genes were found to be enriched for terms such as pathways in cancer, hepatitis B, steroid hormone biosynthesis, arachidonic acid metabolic process, and detoxification of ROS (Table 2).

**Table 2:** MCODE enrichment analysis of the potential targets involved in naringenin treatment of chronic wounds.

MCODE	GO	Description	Log10(*p*)
MCODE_1	hsa05200	Pathways in cancer	− 22.8
MCODE_1	hsa05223	Non-small cell lung cancer	− 20.9
MCODE_1	hsa05212	Pancreatic cancer	− 20.7
MCODE_2	hsa05161	Hepatitis B	− 24.1
MCODE_2	hsa05200	Pathways in cancer	− 23.3
MCODE_2	WP4666	Hepatitis B infection	− 21.9
MCODE_3	hsa00140	Steroid hormone biosynthesis	− 14.5
MCODE_3	GO:0008202	Steroid metabolic process	− 14.1
MCODE_3	GO:0120254	Olefinic compound metabolic process	− 14
MCODE_4	GO:0019369	Arachidonic acid metabolic process	− 10.9
MCODE_4	hsa00590	Arachidonic acid metabolism	− 10.8
MCODE_4	GO:0033559	Unsaturated fatty acid metabolic process	− 9.9
MCODE_5	R-HSA-3299685	Detoxification of reactive oxygen species	− 11.7
MCODE_5	WP3940	One-carbon metabolism and related pathways	− 11
MCODE_5	GO:0098869	Cellular oxidant detoxification	− 10.1

### TF enrichment analysis

The names of 163 target genes were input into the Metascape database to obtain a transcription factor (TF)-target enrichment network. The TF-target enrichment analysis showed that the target genes were regulated by TFs in the TRRUST database (Fig. 3a). In addition, many of the target genes played essential roles in the progression of chronic wound healing as TFs, such as RELA, STAT3, and TP53.

### GO and KEGG pathway enrichment analyses

Packages in the R language were used for GO and KEGG analyses. The Entrez IDs of the 163 target genes are listed in Supplementary Table 2. GO enrichment analyses were conducted to study the biological functions of these target genes in the biological process (BP), molecular function (MF) and cellular component (CC) categories (*P* value < 0.05). The top 12 enrichment results from these analyses were visualized in bubble diagrams (Fig. 3b–d) and histograms (Supplementary Fig. 1a–c). The GO terms enriched in the BP category were mainly the response to oxidative stress, cellular response to chemical stress, response to xenobiotic stimulus, response to nutrient levels, cellular response to oxidative stress, ROS metabolic process, and response to ROS. The important MF terms were the nuclear receptor activity, ligand-activated TF activity, antioxidant activity, heme binding, tetrapyrrole binding, phosphatase binding, peroxidase activity, oxidoreductase activity, and oxygen binding terms. In addition, a vast range of terms related to cellular structures were enriched in the CC category, namely, protein kinase complex, nuclear envelope, basal plasma membrane, basal part of cell, and organelle outer membrane. Based on these results, naringenin was involved in the treatment of chronic wounds by modulating a variety of gene biological functions related to inhibiting oxidative stress, anti-inflammatory activity, and immunoregulatory functions.

A KEGG pathway enrichment analysis was also conducted to better understand the mechanisms of action of the intersecting genes (*P* value < 0.05), and 177 signalling pathways were obtained. The bubble diagram (Fig. 3e) and histogram (Supplementary Fig. 1d) showed the top 12 signalling pathways, many of which were closely associated with a variety of chronic wounds, such as apoptosis^70^, HIF-1 signalling pathways^71^ and ROS^72^. Additionally, the AGE-RAGE signalling pathway in diabetic complications^73^ was involved in diabetic wound healing; hepatitis B^74^, human cytomegalovirus infection and hepatitis C^75^ signalling pathways might participate in chronic infectious wounds^76^. As shown in Fig. 3f, the correlations of “targets to pathways” were visualized, and a network diagram was constructed. The connection between the crucial pathways and the common targets of naringenin suggested that naringenin might treat chronic wounds by acting on multiple pathways and multiple targets. Among them, RELA^77, 78^, AKT1^79, 80^, MAPK1^81–83^ and MAPK3^81–83^are targets closely related to apoptosis, HIF-1 signalling pathways and ROS, which are considered to participate in various chronic wounds.

### Molecular docking

**Figure 4:**
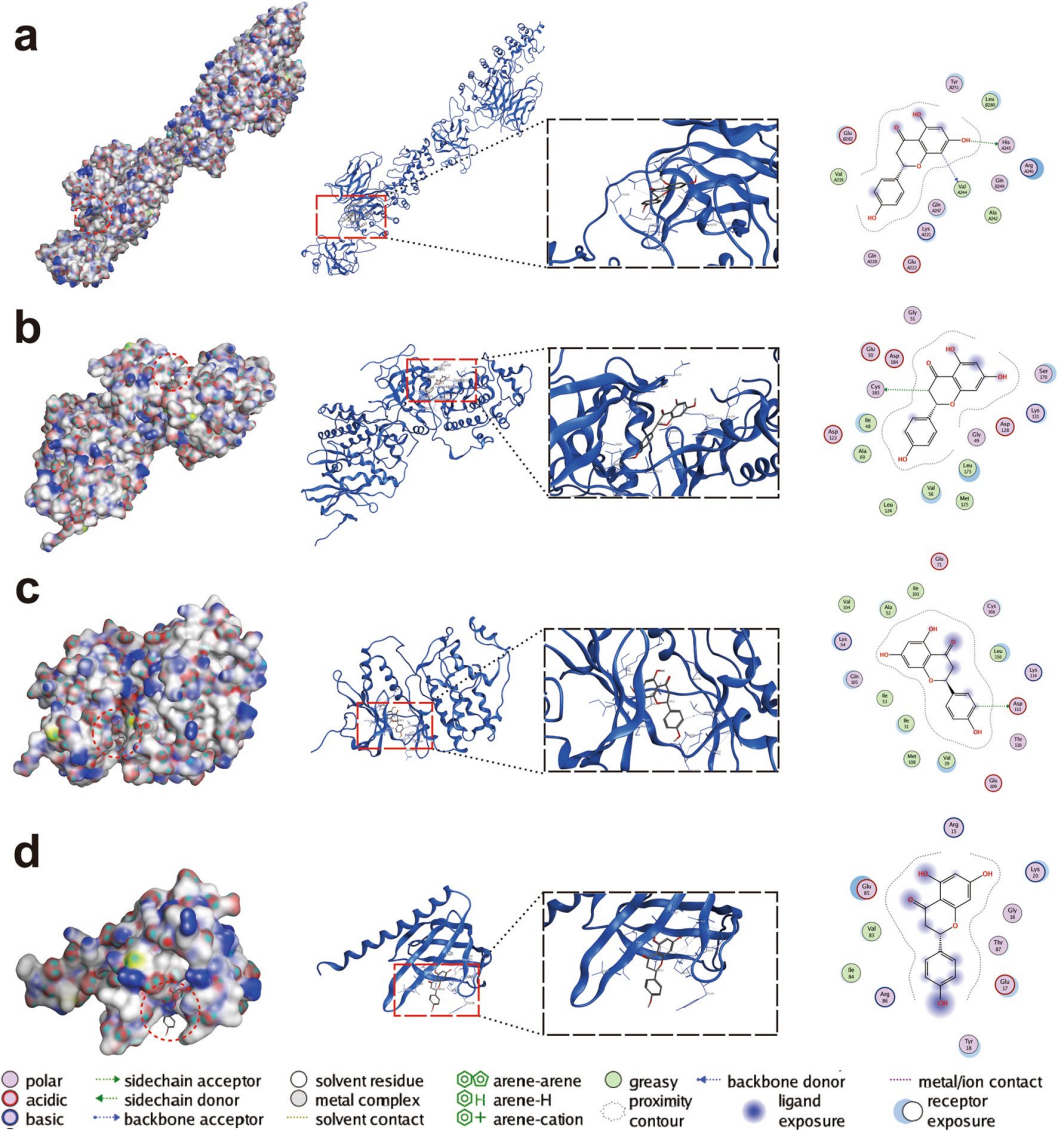
Molecular docking of the key targets that bind to naringenin. The binding poses of naringenin with (**a**) RELA, (**b**) MAPK3, (**c**) MAPK1 and (**d**) AKT1 are shown.

Molecular docking was performed to detect the binding capacity and the interaction modes between naringenin and key target proteins (RELA, AKT1, MAPK1 and MAPK3). The ball-and-stick model and cartoon chain represent the naringenin molecule and a protein, respectively. The lower the vina scores, the more stable the binding between naringenin and the protein, indicating a stronger interaction between naringenin and the receptor. The results verified that the four targets all stably bound to naringenin. The lowest to highest vina scores for the target proteins were RELA, MAPK3, MAPK1 and AKT1 (Table 3). A 3D map of naringenin binding to the target proteins was shown in Fig. 4.

**Table 3:** Molecular docking parameters and results for the 4 targets that bind to naringenin.

No.	Protein name	Vina scores (kcal/mol)	Cavity size	Center^a^			Size^b^		
				X	Y	Z	Z	Y	Z
1	RELA	− 9.2	3683	− 3.33	78.834	106.313	32	21	21
2	MAPK3	− 8.7	4384	24.835	− 1.787	18.102	21	31	34
3	MAPK1	− 6.5	1966	32.129	− 3.237	− 9.653	21	21	21
4	AKT1	− 6.4	303	3.736	− 5.635	7.019	21	21	21

^a^Docking pocket center coordinates. Unit: angstroms.

^b^Size of the docking pocket in the X, Y, and Z directions. Unit: angstroms.

### Naringenin promotes skin wound healing in mice

**Figure 5:**
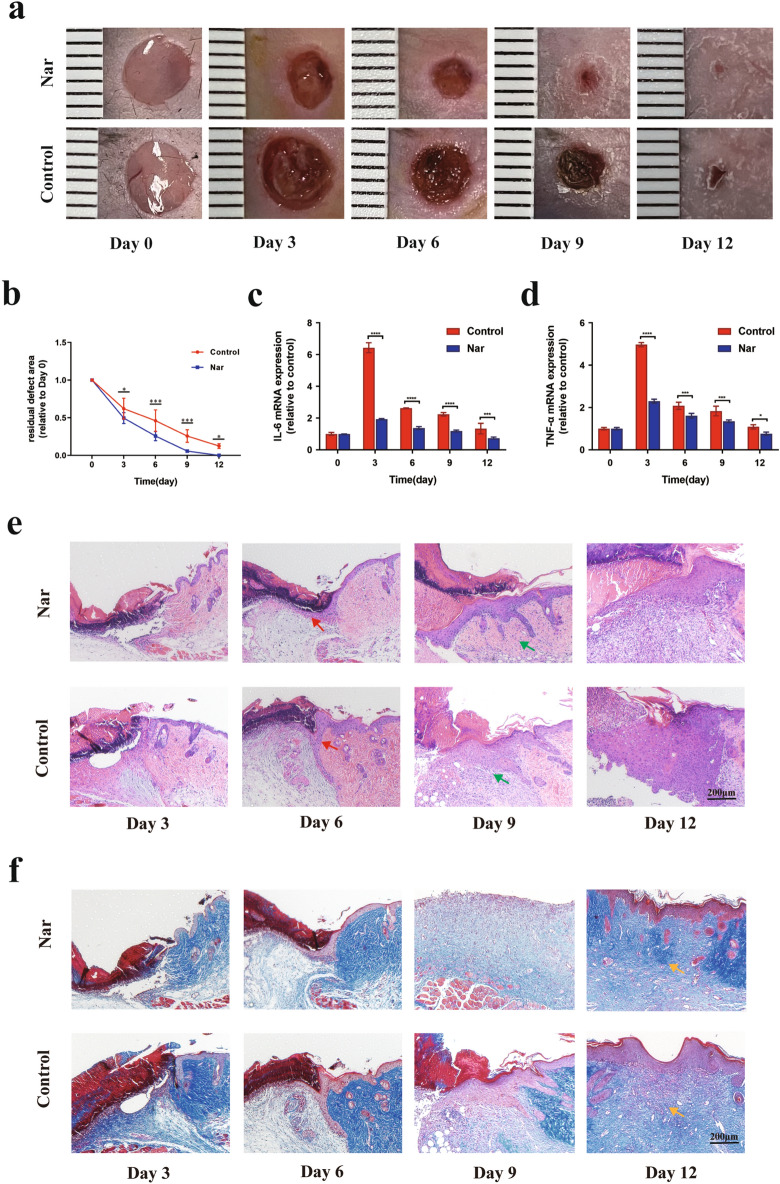
Effects of naringenin on wound healing. (**a**) Representative images of skin wounds in mouse models. Unit: millimetres (mm) (**b**) Residual defect areas. (**c**,**d**) The mRNA expression of inflammation-related markers in mice. Representative images of H&E (**e**) and Masson’s trichrome staining (**f**) of the sections on days 3, 6, 9 and 12 post-wounding in the naringenin-treatment group and control group, scale bar = 200 µm. Red arrows indicate the formation of new epithelial tissue, green arrows indicate inflammatory cells, and yellow arrows indicate collagen fiber. Data are presented as the means ± SDs of 5–6 mice. Bar chart data were compared by Student’s t test or ANOVA. *P < 0.05, **P < 0.01, ***P < 0.001, and ****P < 0.0001.

Generally, chronic wounds are caused by persistent inflammation and are unable to transition from the inflammatory phase to the proliferative phase^2^. We tested the function of naringenin in mouse skin wounds and focused on the inflammatory status by examining the expression of proinflammatory cytokines at the edge of the wound to validate the therapeutic effect of naringenin on skin wound healing. Combined with the residual defect area of wounds on both sides of the backs of mice, we found that the wound healing was significantly accelerated in mice from the naringenin-treatment group compared with the control group (Fig. 5a,b). TNF-α and IL-6, which are potential targets of naringenin shown in Fig. 2d, are proinflammatory cytokines with increased expression in the wound microenvironment^84^. As shown in Fig. 5c,d, the mRNA levels of TNF-α and IL-6 in wound tissue were significantly higher than those in normal tissue. Meanwhile, naringenin effectively alleviated wound inflammation characterized by reducing the expression of the TNF-α and IL-6 mRNAs. H&E staining and Masson trichrome staining were performed at different time points to show inflammation and the formation of granulation tissue during the wound healing process between control group and naringenin-treatment group (Fig. 5e,f). H&E staining showed that, compared with the control group, the number of inflammatory cells in skin wounds of mice in the Naringenin-treatment group decreased on day 3. A certain thickness of new epithelium began to form at 6 days, and at 9 days and 12 days, the epidermis of Naringenin-treatment group became thinner and migrated well. Masson's trichromatic staining showed that the wound bed was filled with granulation tissue on day 12 after wounding, and collagen fiber configurations were more regular on days 9 and 12 in the naringenin-treatment group compared with the control group.

### Effect of naringenin on the viability of HaCaT cells and HFFs

**Figure 6:**
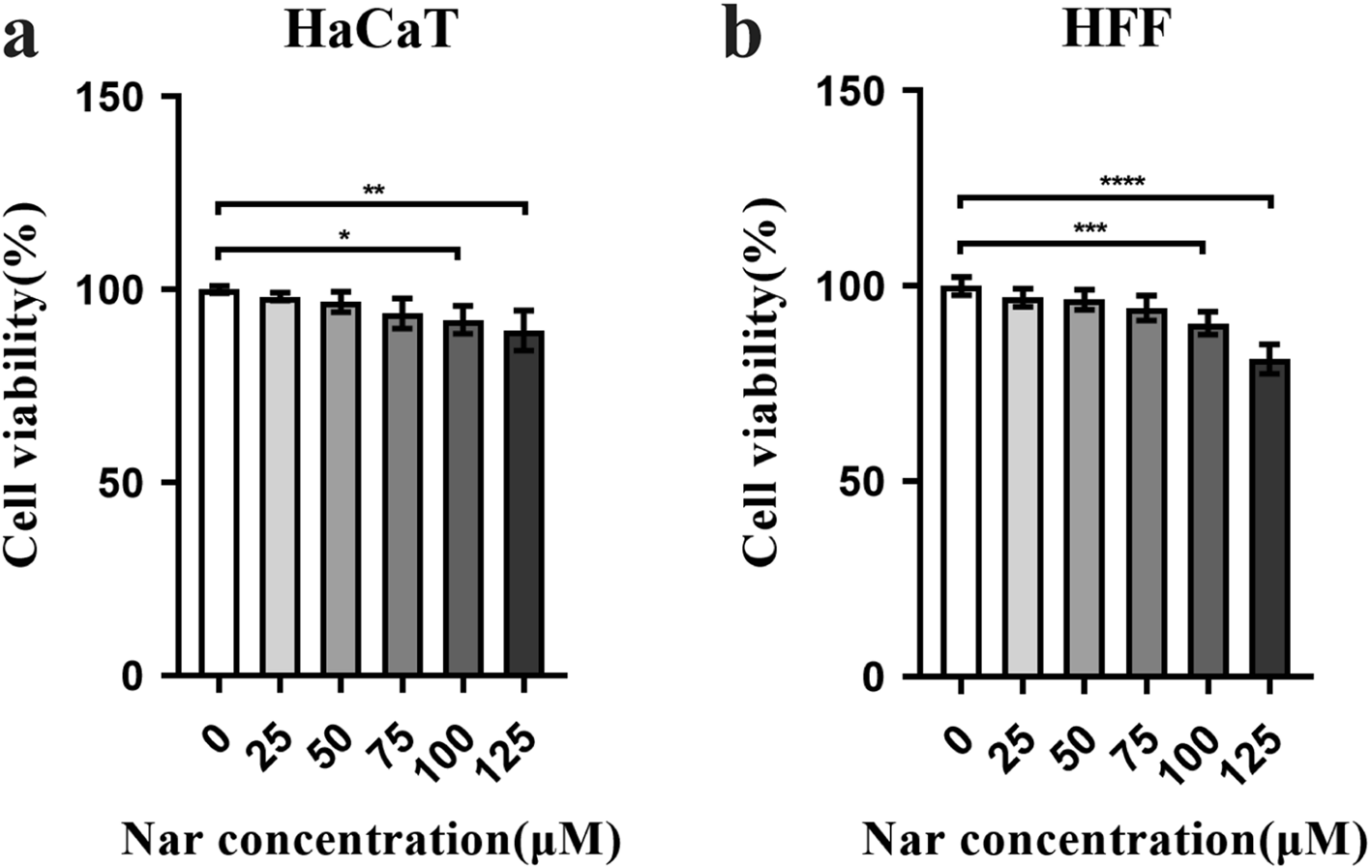
Effect of naringenin on the viability of HaCaT cells (**a**) and HFFs (**b**). After treatment with naringenin for 24 h, the viability of these cells was measured. For all studies, n ≥ 3. Error bars show the means ± SDs. Bar chart data were compared by ANOVA. *P < 0.05, **P < 0.01, ***P < 0.001, and ****P < 0.0001.

Based on the results of in vivo experiments, as well as the concentration at which naringenin has been shown to exert effects in other studies^42, 85, 86^, we designed different concentration gradients for cell viability assays. The CCK-8 assay has been used to determine the number of viable cells in cell proliferation or toxicity assays based on the principle that WST-8 can be reduced to water-soluble formazan dyes by some dehydrogenases in mitochondria in the presence of electron carrier 1-methoxy PMS. As shown in Fig. 6, different concentrations (0 µM, 25 µM, 50 µM, 75 µM, 100 µM and 125 µM) of naringenin exerted different effects on the proliferation of HaCaT cells and HFFs, which were immortalized epidermal keratinocytes and dermal fibroblasts, respectively. In the low concentration range, naringenin did not exhibit obvious cytotoxicity towards HaCaT cells and HFFs. As the concentrations of naringenin increased, the viability of both cell types decreased.

### Effect of naringenin on apoptosis in HaCaT cells and HFFs

**Figure 7:**
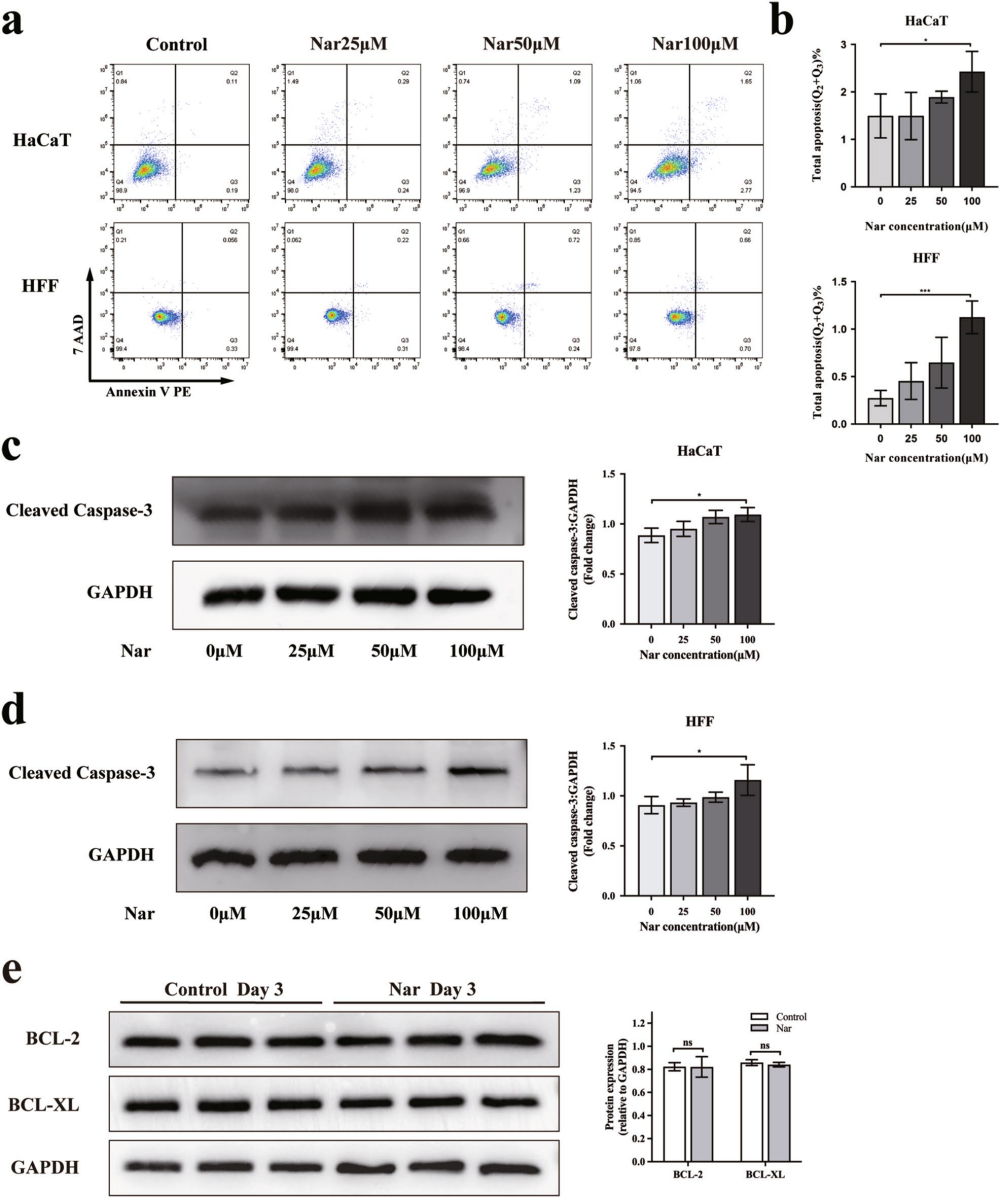
Effect of naringenin on cell apoptosis. HaCaT cells and HFFs were treated with naringenin for 3 h. (**a**) HaCaT cells and HFFs were stained with PE-conjugated Annexin V and 7-AAD, and the apoptosis rate (**b**) of cells was analysed. Representative Western blots showing the changes in cleaved caspase 3 protein expression in HaCaT cells(**c**) and HFFs(**d**) normalized to GAPDH. (**f**) Representative bands of BCL-2, BCL-XL protein levels in the wound tissue of mice on day 3 were detected by Western blotting. Original blots are presented in Supplementary Fig. 4. For all studies n ≥ 3. Error bars show the means ± SDs. Bar chart data were compared by ANOVA. *P < 0.05, **P < 0.01, ***P < 0.001, and ****P < 0.0001.

According to the KEGG enrichment analysis in Fig. 3e, apoptosis is involved in naringenin treatment of chronic wounds. Annexin V is a 35–36 kDa Ca^2+^-dependent phospholipid binding protein with high affinity for the membrane phospholipid phosphatidyl serine (PS) and binds to PS exposed early in apoptosis. 7-Amino actinomycin D (7-AAD) is a nonmembrane permeable fluorescent indicator that can be embedded in nucleic acids. PE Annexin V staining was used in conjunction with 7-AAD to identify cells at different stages of apoptosis. HaCaT cells and HFFs were treated with naringenin for 3 h to confirm the effect of naringenin on apoptosis in skin tissue. As shown in Fig. 7a,b, a low concentration of naringenin had little effect on cell apoptosis. However, with the increase of concentration, naringenin could induce cell apoptosis (*P* < 0.05). Notably, the apoptosis-related protein CASP3 was a potential target of naringenin in Fig. 2d. Consistently, with the increase in naringenin concentration, the expression of the cleaved caspase 3 protein in HaCaT cells and HFFs gradually increased (Fig. 7c,d). On day 3, there was no significant difference in the protein levels of BCL-2 and BCL-XL in the wound tissue between the control group and the naringenin-treatment group (Fig. 7f). Collectively, the results indicated that cell apoptosis might be involved in the alteration of cell viability by high concentrations of naringenin.

### Naringenin attenuates LPS-induced ROS production in HaCaT cells and HFFs

**Figure 8:**
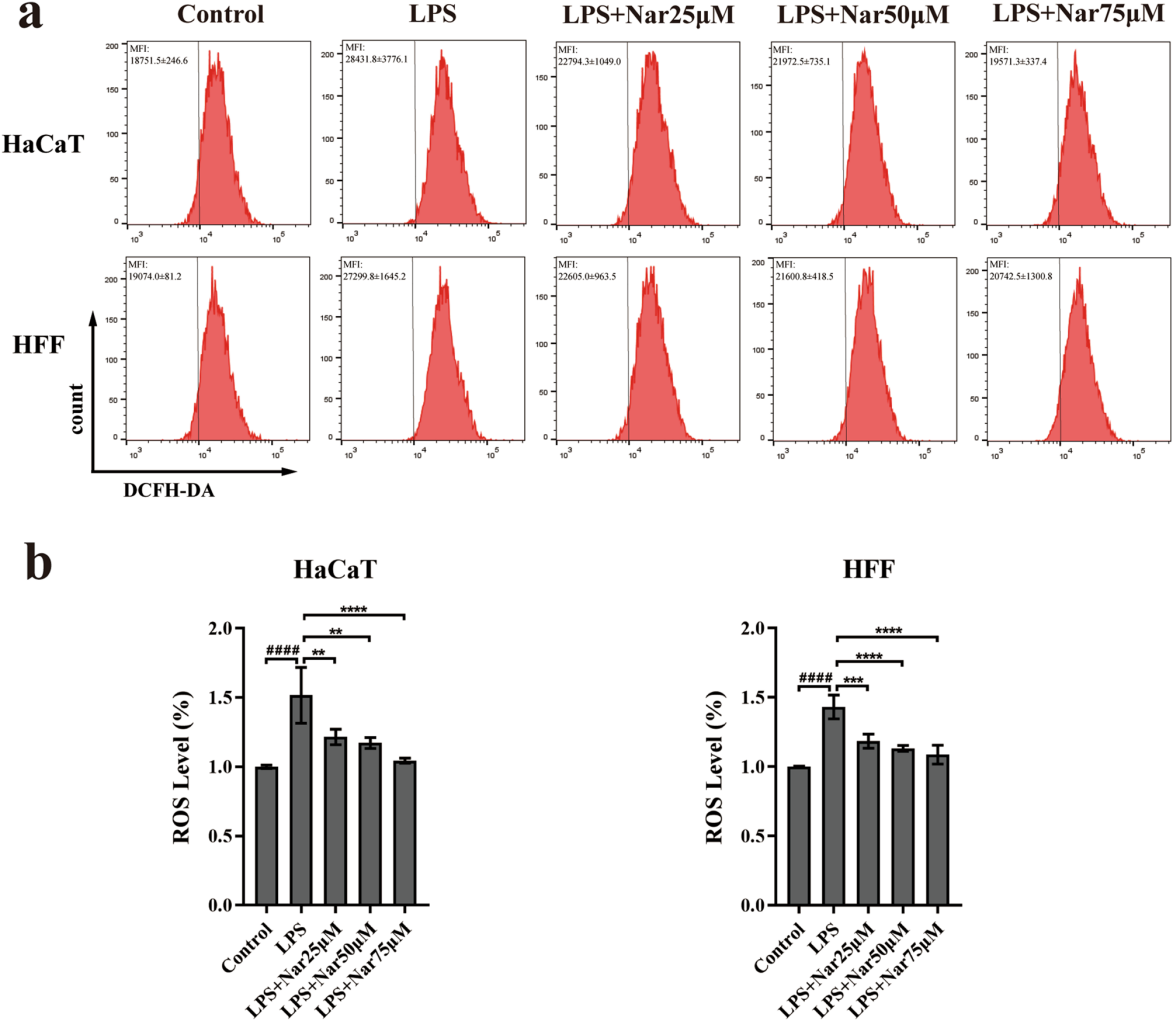
Effect of naringenin on intracellular ROS production. (**a**) HaCaT cells and HFFs were pretreated with (or without) naringenin (25 µM, 50 µM, or 75 µM) for 3 h and then exposed to LPS (0.5 µg/ml) for 12 h. Fluorescence was detected using flow cytometry. (**b**) Changes in ROS levels. For all studies n ≥ 3. Error bars show the means ± SDs. Bar chart data were compared by ANOVA. ^####^*P* < 0.0001 compared with the control group, and *P < 0.05, **P < 0.01, ***P < 0.001, and ****P < 0.0001 compared with the LPS group.

Oxidative stress is characterized by the overproduction of ROS^87^. Oxidative stress often occurs during a period of persistent inflammation, causing cell and tissue damage and contributing directly or indirectly to disease progression^88^. Meanwhile, oxidative stress was identified to participate in the biological process of naringenin treatment of chronic wounds, as shown in Fig. 3b. DCFH-DA can be hydrolysed by intracellular esterase to produce DCFH. Intracellular reactive oxygen species can oxidize non-fluorescent DCFH to generate fluorescent DCF. The fluorescence intensity of DCF can reflect the level of intracellular ROS. We measured the fluorescence intensity of DCF after treatment with naringenin to verify the key role of oxidative stress in the effects of naringenin on chronic wounds. LPS-treated cells were used to simulate the oxidative stress state of wound cells^89^. We treated HaCaT cells and HFFs with non-cytotoxic concentrations of naringenin before priming the cells with LPS (0.5 µg/ml, 12 h). ROS levels in HaCaT cells and HFFs were significantly increased after LPS stimulation, as shown in Fig. 8. However, the LPS-induced increase in ROS levels was diminished by naringenin in HaCaT cells and HFFs in a dose-dependent manner.

### Naringenin inhibits proinflammatory cytokine production in LPS-treated HaCaT cells and HFFs

**Figure 9:**
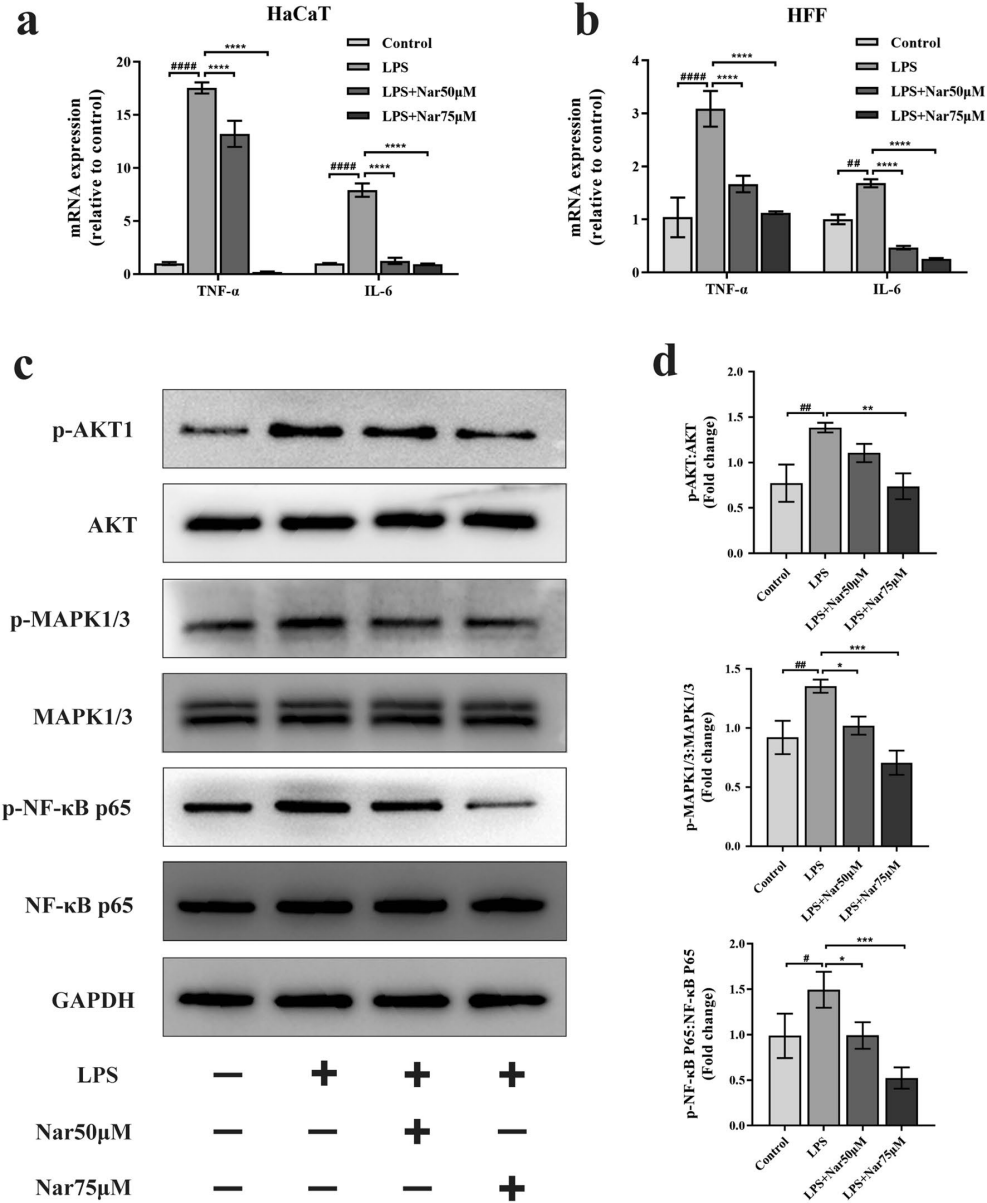
Naringenin inhibits LPS-induced inflammation and regulates the AKT/MAPK/NF-κB p65 signalling pathway. HaCaT cells and HFFs were pretreated with (or without) naringenin (25 µM, 50 µM, or 75 µM) for 3 h and then exposed to LPS (0.5 µg/ml) for 12 h. Naringenin inhibited the mRNA expression of TNF-α and IL-6 in LPS-treated HaCaT cells (**a**) and HFFs (**b**). (**c**) Representative bands of AKT1, MAPK1/3 and NF-κB p65 phosphorylation in HaCaT cells were detected by Western blotting. (**d**) Quantitation of the corresponding protein levels. Original blots are presented in Supplementary Fig. 4. For all studies, n ≥ 3. Error bars show the means ± SDs. Bar chart data were compared by ANOVA. ^#^*P* < 0.05, ^##^*P* < 0.01 and ^####^*P* < 0.0001 compared with the control group, and *P < 0.05, **P < 0.01, ***P < 0.001, and ****P < 0.001 compared with the LPS group.

Dysregulated inflammation is an important mechanism contributing to the development of chronic wounds^90^. Here, we evaluated whether naringenin eliminated LPS-induced inflammation by measuring the mRNA expression of proinflammatory cytokines in HaCaT cells and HFFs. As expected, LPS treatment significantly increased the levels of TNF-α and IL-6 in both cell types, while cells pretreated with 50 µM and 75 µM naringenin displayed markedly decreased expression of the corresponding genes in a dose-dependent manner (Fig. 9a,b).

### Naringenin regulates the AKT, MAPK1/3 and NF-κB p65 signalling pathways

Based on the “targets-pathways” network shown in Fig. 3f, RELA, AKT1, MAPK1 and MAPK3 were identified and predicted to be involved in the regulation of chronic wound healing. Previous studies have indicated that the NF-κB p65/RELA, AKT1, MAPK1 and MAPK3 signalling pathways are involved not only in ROS production but also in inflammation^91–94^. Western blot analysis was performed to further explore the participating signalling pathways and identify the anti-inflammatory and antioxidant mechanisms of naringenin in HaCaT cells and HFFs. As shown in Fig. 9c,d, p-AKT1, p-MAPK1/3, p- NF-κB p65/RELA levels were noticeably increased in the LPS group compared with the levels in the control group in HaCaT cells. However, the levels of the phosphorylated AKT1, MAPK1/3, NF-κB p65/RELA proteins in the naringenin group were obviously lower than those in the LPS group in HaCaT cells. Representative bands of AKT1, MAPK1/3, and NF-κB p65 phosphorylation levels in HFFs detected by Western blotting are provided in Supplementary Fig. 2. AKT1, MAPK1/3, and NF-κB p65 phosphorylation levels in the wound tissue of mice on day 3 detected by Western blotting are provided in Supplementary Fig. 3. Taken together, these findings indicated that naringenin exerts anti-inflammatory and antioxidant effects by inhibiting the AKT1/MAPK/RELA signalling pathway.

## Discussion

Chronic wounds are the most common skin disease, especially in individuals who are elderly, are diagnosed with diabetes, or have vascular diseases^95^. Chronic wounds typically present with persistent inflammation, bacterial colonization, impaired reepithelization, angiogenesis, and excessive ROS levels^96, 97^. Flavonoids are a large group of plant-derived compounds with a wide variety of biological functions, and certain flavonoids, such as naringenin, are well-established to exert therapeutic effects on skin disorders^98^. Although one study has shown that naringin has the potential to promote acute wound healing in rats^99^, no studies have elucidated the related molecular mechanism by which naringenin treats chronic wounds. The use of systematic network pharmacology for the identification and optimization of screening biotargets, biological processes, functional pathways and binding capacities is a meaningful approach for research on the effects of naringenin on treating chronic wounds. The therapeutic effect and mechanism of naringenin were verified in vivo and in vitro in the current study, confirming that naringenin exhibits potent pharmacological activity and shows promise for the treatment of chronic wounds.

Although the exact aetiology of chronic skin wounds is still uncertain, chronic wounds are recognized as a chronic disease linked to the inflammatory response and oxidative stress^100, 101^. The removal of oxygen free radicals and the inhibition of inflammation play crucial roles in healing chronic wounds^102^. Oxidative stress and inflammation are interrelated processes that coexist in an inflammatory environment^103^. Excessive ROS levels are considered important oxidative stress and proinflammatory mediators^104^. The bioinformatics analysis performed in this study yielded 163 potential biotargets of naringenin in treating chronic wounds. According to the GO enrichment analysis, these targets were mainly involved in oxidative stress, inflammation and metabolic processes. Moreover, the MCODE enrichment analysis showed that the potential biotargets were related to cancer, infection, metabolism and oxidative stress-associated diseases. Consistent with the MCODE enrichment results, the KEGG enrichment analysis also revealed a diverse set of pathways related to cancer, infection, metabolism and oxidative stress. Because cancer affects many biological processes and targets and many of these targets are involved in cell proliferation and survival, cell viability assays were performed to examine whether naringenin affects cell proliferation and survival. Among the top 12 signalling pathways screened by KEGG, apoptosis can also affect cell survival, so we performed cell apoptosis assay. In vitro experiments showed that low-dose naringenin had no significant effect on normal skin cell viability and cell apoptosis, and the same concentration of naringenin significantly decreased intracellular ROS production and inhibited the expression of the inflammatory cytokines TNF-α and IL-6. TNF-α, as a main regulator of proinflammatory cytokines, induces an inflammatory response and the development of chronic inflammatory diseases^105^. IL-6 plays different roles in the inflammatory response^106, 107^. In chronic stress, early trauma, and acute inflammation caused by infection, the expression of IL-6, a proinflammatory biomarker, is increased along with various putative biomarkers of inflammation, including TNF-α and CRP^106^. High naringenin concentrations were cytotoxic, and cell apoptosis was speculated to be one of the possible pathways activated by naringenin. However, 100 μM naringenin promoted wound healing in mice, and the authors suggested that the wound microenvironment partially weakened the cytotoxicity caused by naringenin-induced apoptosis. Meanwhile, naringenin alleviated wound inflammation by reducing the secretion of inflammatory factors in a mouse wound model. These results indicated that different concentrations of naringenin exerted different effects on cell survival, and low concentrations of naringenin promoted wound healing by alleviating oxidative stress and reducing the inflammatory response.

Based on the “targets-pathways” network, we screened and identified AKT1, RELA, MAPK1 and MAPK3 as potential key targets. According to the PPI network, the target proteins were not independent of each other but rather were connected and interacted with each other^108^. The molecular docking experiment indicated that naringenin could bind tightly to these screened key target proteins. The endotoxin lipopolysaccharide (LPS) is a component of gram-negative bacteria and is widely used to simulate the inflammatory and oxidative stress state of wound healing in vitro^89, 109, 110^. LPS binds to Toll-like receptor 4 (TLR4) and activates multiple signalling pathways through signal transduction, including NF-κB p65/RELA and MAPKs, ultimately resulting in increased transcription of the proinflammatory cytokines TNF-α and IL-6^111^. Furthermore, our current study revealed that LPS strongly activated the AKT1, MAPK1/3 and NF-κB p65/RELA pathways and increased TNF-α and IL-6 expression. The MAPK family includes c-Jun NH_2_-terminal kinase (JNK), protein 38 and extracellular signal-regulated kinase (ERK1, also known as MAPK3; ERK2, also known as MAPK1) and is responsible for the expression of proinflammatory cytokines^112^. Meanwhile, MAPKs also participate in regulating NF-κB p65/RELA transcriptional activity^113^. NF-κB p65/RELA, a member of the inducible transcription factor family, is involved in different inflammatory processes and controls the expression of the inflammatory genes TNF-α and IL-6 through a noncanonical pathway^114^. The TF-target enrichment analysis showed that RELA was a critical TF involved in the effects of naringenin on chronic wounds. AKT1, an upstream molecule of NF-κB p65/RELA, is involved in regulating oxidative stress and the inflammatory response through the NF-κB pathway^91^. Western blotting proved that naringenin certainly suppressed the LPS-induced phosphorylation of AKT1, NF-κB p65/RELA and MAPK1/3. Meanwhile, compared with the control group, the phosphorylation levels of AKT1, MAPK1/3, and NF-κB p65 in the wound tissue of mice in the naringenin-treatment group were significantly decreased. A reasonable speculation is that naringenin inhibits inflammation and oxidative stress in the treatment of chronic wounds by regulating the AKT1, RELA and MAPK1/3 signalling pathways.

A limitation of using naringenin is that high concentrations of naringenin are cytotoxic and may affect the healing of chronic wounds. Therefore, the optimal naringenin concentration for the treatment of chronic wounds must be further determined. Recently, studies on chronic wounds have mostly focused on the application of various new materials as wound dressings to improve chronic wound healing. Naringenin might be used as one of the components of a new wound dressing for wound healing; however, its physicochemical characteristics and solubility must be improved.

In conclusion, comprehensive network pharmacology, molecular docking and in vivo and in vitro experiments have shown that naringenin potentially treats chronic wounds by alleviating oxidative stress and reducing the inflammatory response. The underlying mechanism of naringenin in chronic wound therapy involves the inhibition of ROS production and the expression of inflammatory cytokines by regulating the AKT1, RELA and MAPK1/3 signalling pathways. These findings reveal the molecular biological mechanism of naringenin in the treatment of chronic wounds, enrich the range of pharmacological applications of naringenin and lay a foundation for further clinical applications.

## Supplementary Information

Supplementary Information.

## Data Availability

The datasets presented in this study are available in online repositories. All data generated or analysed during this study can be obtained upon reasonable request to the corresponding author.
