# Supplementary material for: Integrated network pharmacology and experimental validation to explore the mechanisms underlying naringenin treatment of chronic wounds
Source: Sci Rep. 2023 Jan 4;13:132. doi: 10.1038/s41598-022-26043-y (PMC9811895; doi:10.1038/s41598-022-26043-y)
Supplement: Supplementary file 1 — Supplementary Information. [file 41598_2022_26043_MOESM1_ESM.pdf]

Supplementary Materials

Integrated Network Pharmacology and Experimental Validation to Explore the Mechanisms Underlying Naringenin Treatment of Chronic Wounds

Rui Sun[1,4], Chunyan Liu[2,4], Jian Liu[1,2,4], Siyuan Yin[1,2,4], Ru Song[1,4], Jiaxu Ma[1,4], Guoqi Cao[1,4], Yongpan Lu[3,4], Guang Zhang[1,4], Zhenjie Wu[1,4], Aoyu Chen[2,4], and Yibing Wang[1,2,3,4*]

[1]Department of Plastic Surgery, Shandong Provincial Qianfoshan Hospital, Shandong University, Jinan, Shandong, 250012, P. R. China.

[2]Department of Plastic Surgery, The First Affiliated Hospital of Shandong First Medical University & Shandong Provincial Qianfoshan Hospital, Jinan, Shandong, 250014, P. R. China.

[3]The First Clinical Medical College, Shandong University of Traditional Chinese Medicine, Jinan, Shandong, 250014, P. R. China

[4]Jinan Clinical Research Center for Tissue Engineering Skin Regeneration and Wound Repair, Jinan, Shandong, 250014, P. R. China [*]Correspondence:

Yibing Wang

Email: ybwang@sdfmu.edu.cn.

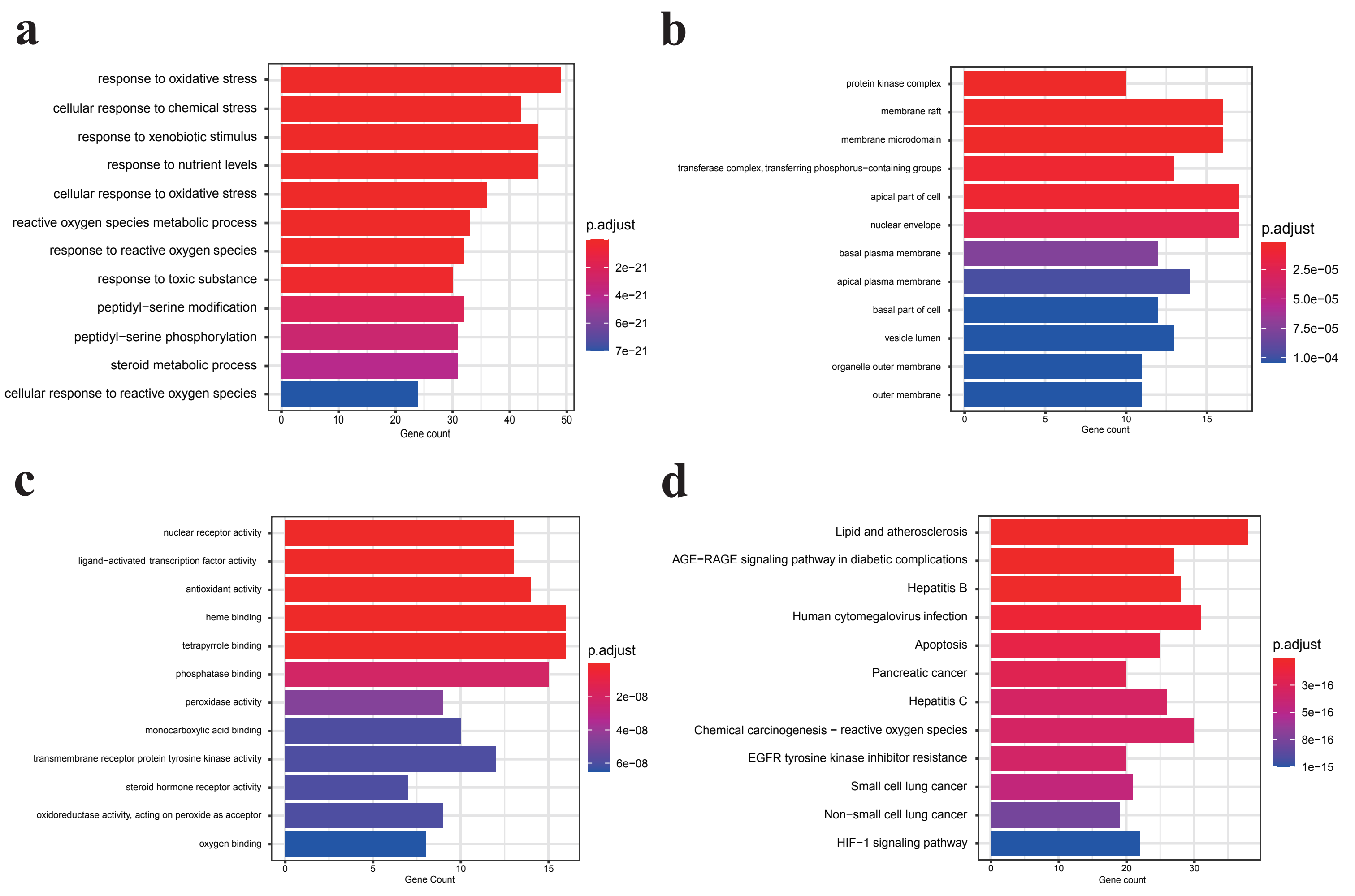

Supplementary Figure 1 The histograms show GO and KEGG enrichment analysis of naringenin against chronic wound. The (A)biological process, (B) cellular component (C) molecular function of GO analysis and the (D) molecular pathways of 163 intersecting genes for naringenin treating chronic wound.

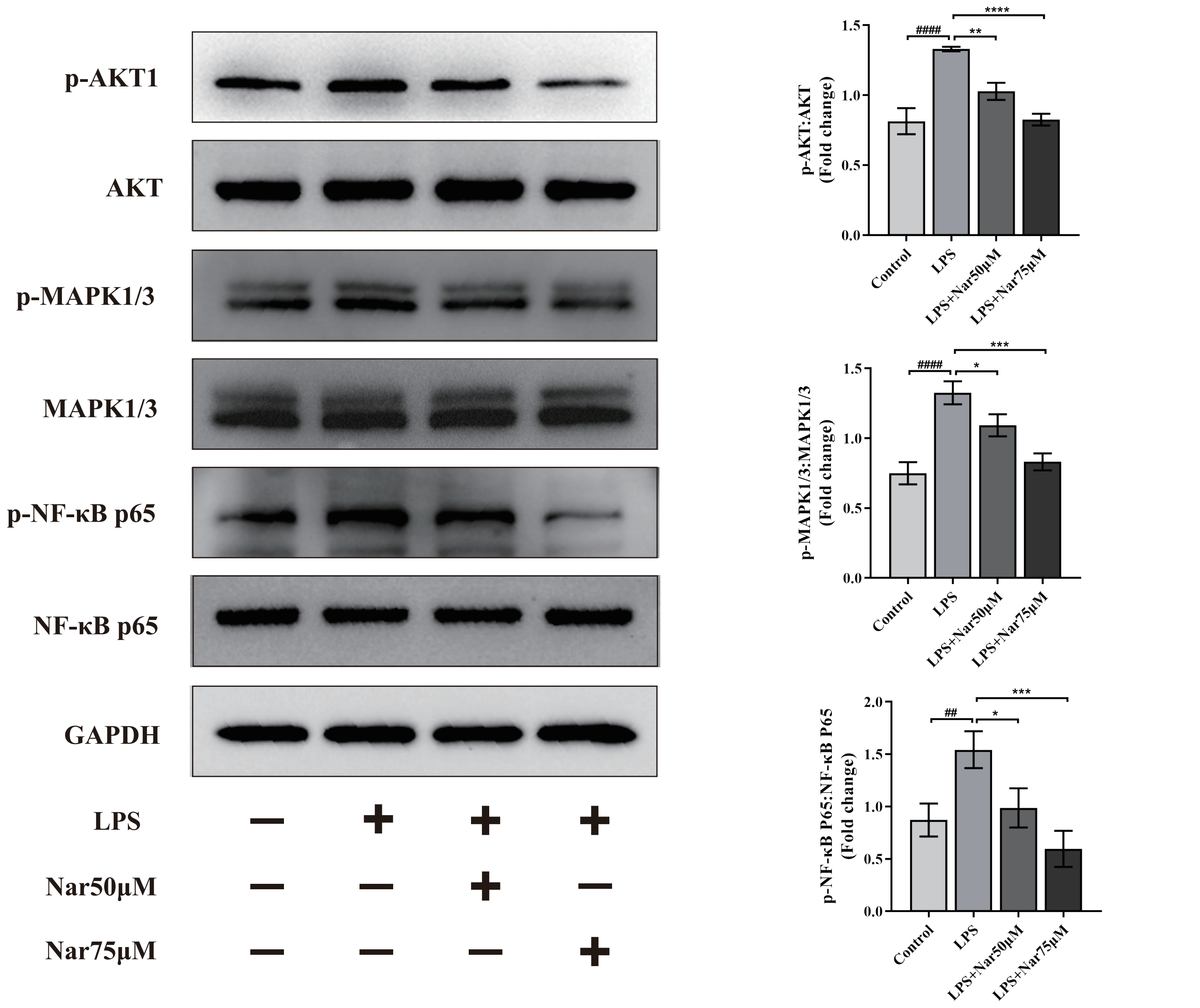

Supplementary Figure 2 Representative bands of AKT1, MAPK1/3, and NF-κB p65 phosphorylation levels in HFFs were detected by Western blotting

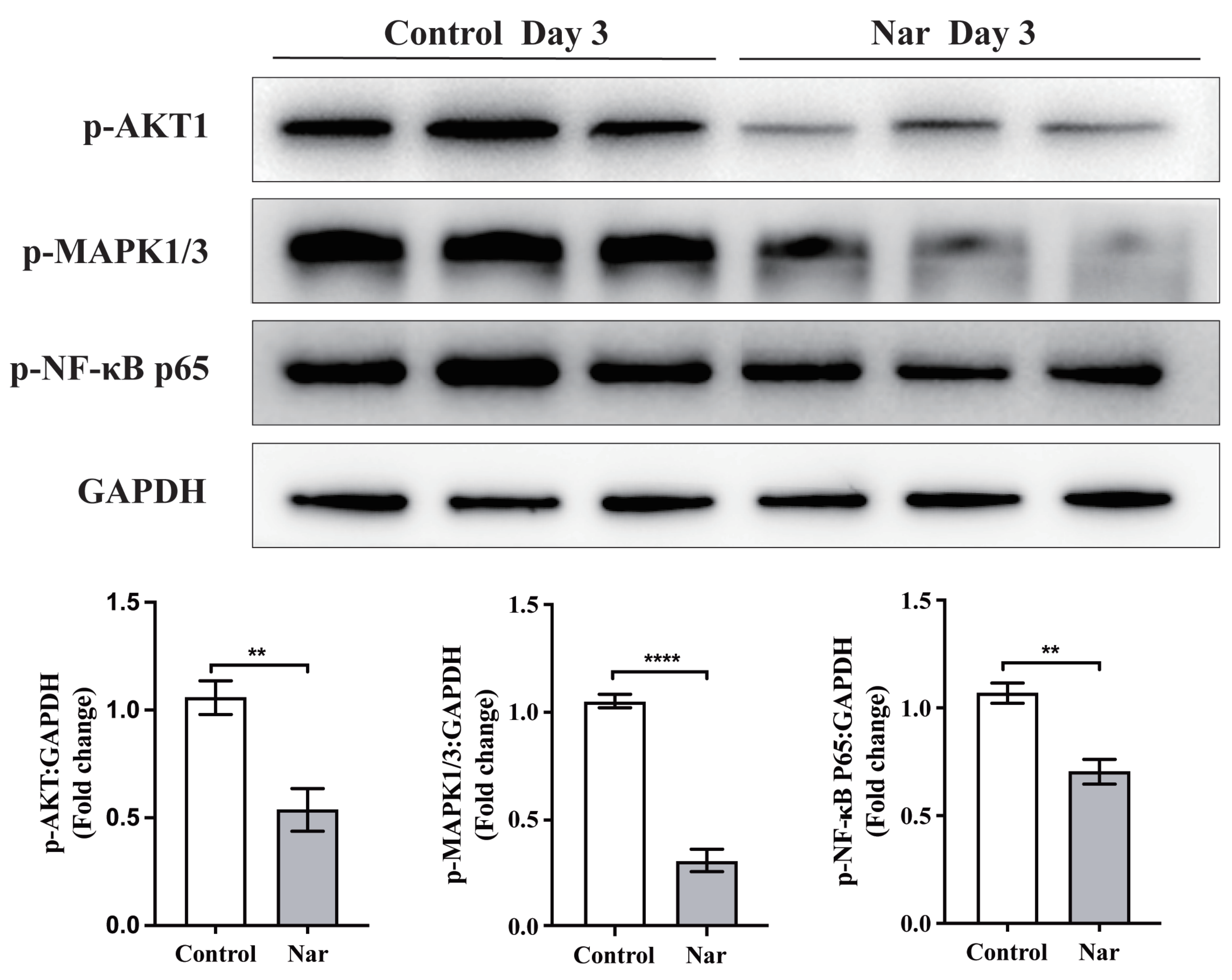

Supplementary Figure 3 Representative bands of AKT1, MAPK1/3, and NF-κB p65 phosphorylation levels in the wound tissue of mice on day 3 were detected by Western blotting

Original images of Figure 1 and Figure 7

HaCaT

CLEAVED CASPASE 3 : 17/19 kDa

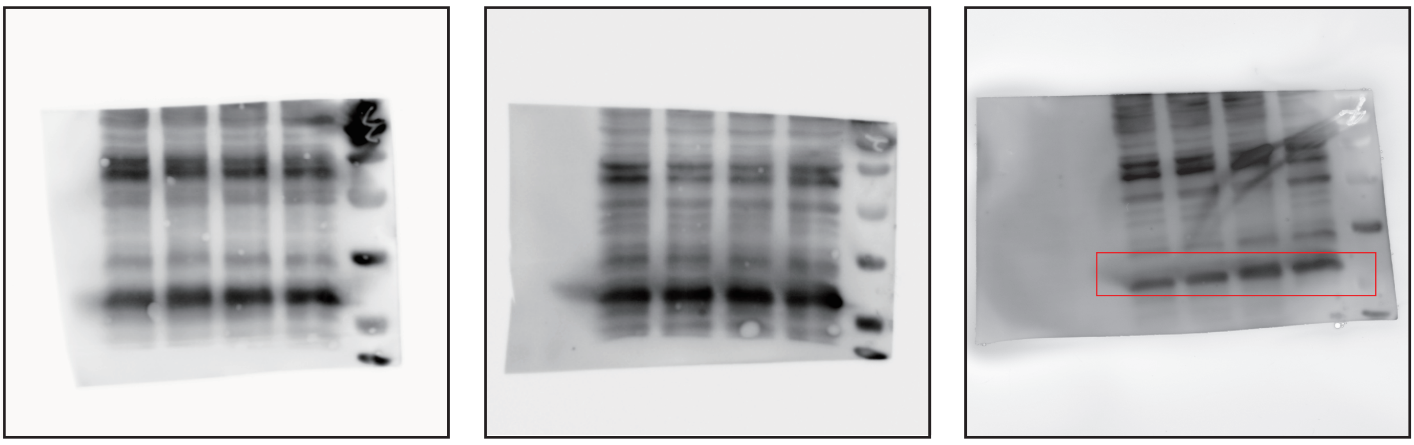

GAPDH : 36 kDa

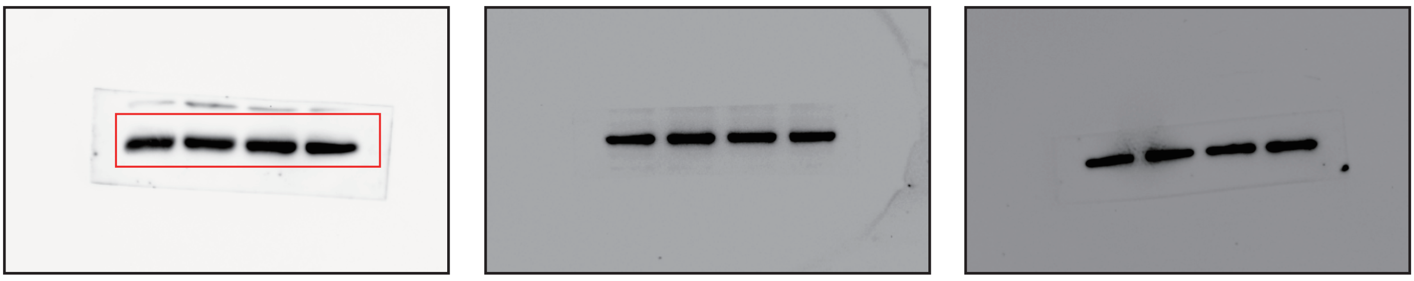

HFF

CLEAVED CASPASE 3 : 17/19 kDa

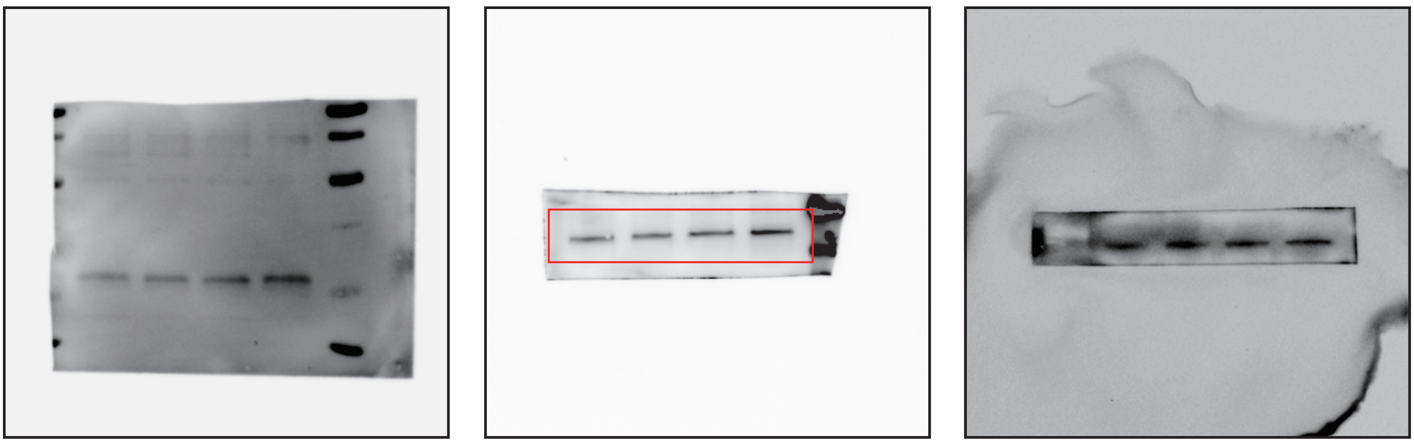

GAPDH : 36 kDa

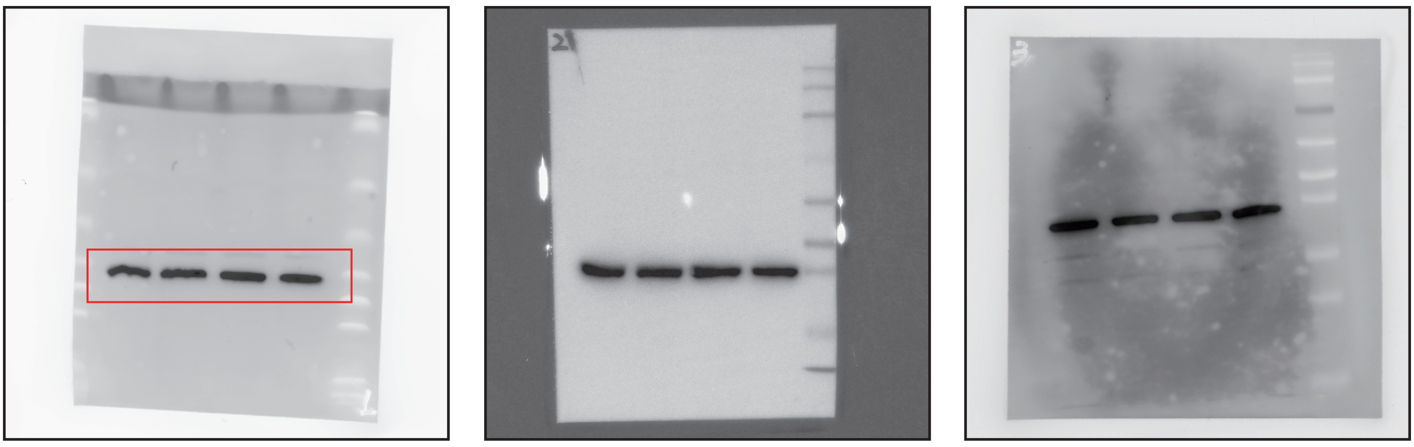

Wound tissue of mice on day 3

BCL-2 : 26 kDa

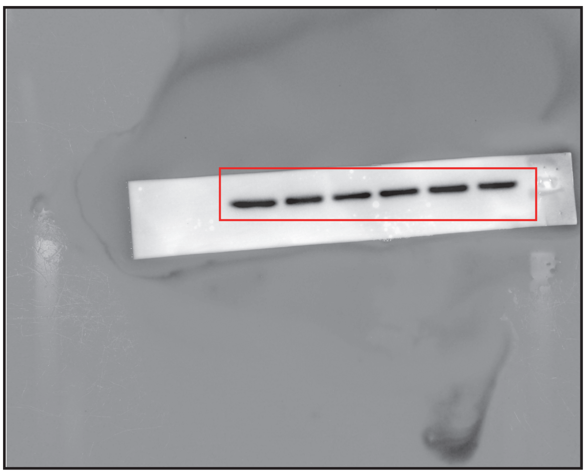

BCL-XL : 30 kDa

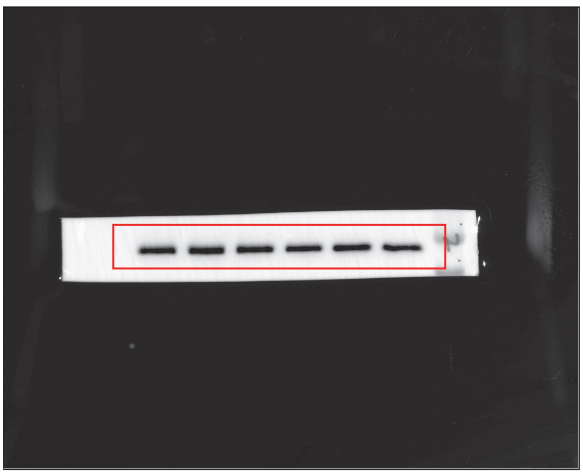

GAPDH : 36 kDa

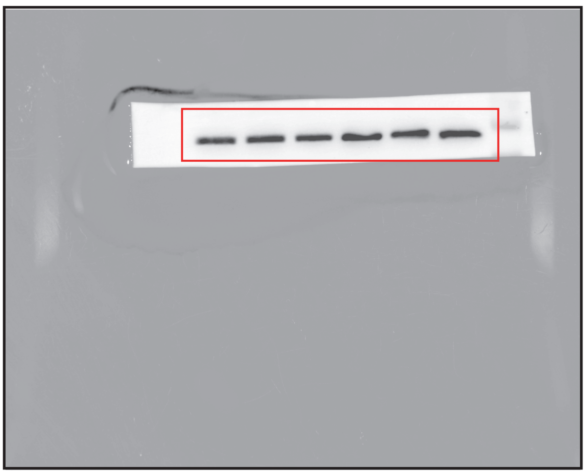

Original images of Figure 9

HaCaT

p-AKT1 : 60 kDa

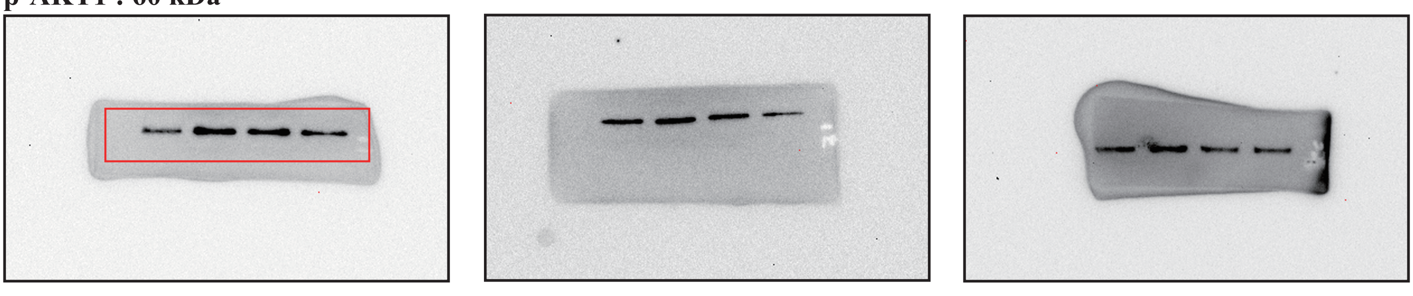

AKT : 60 kDa

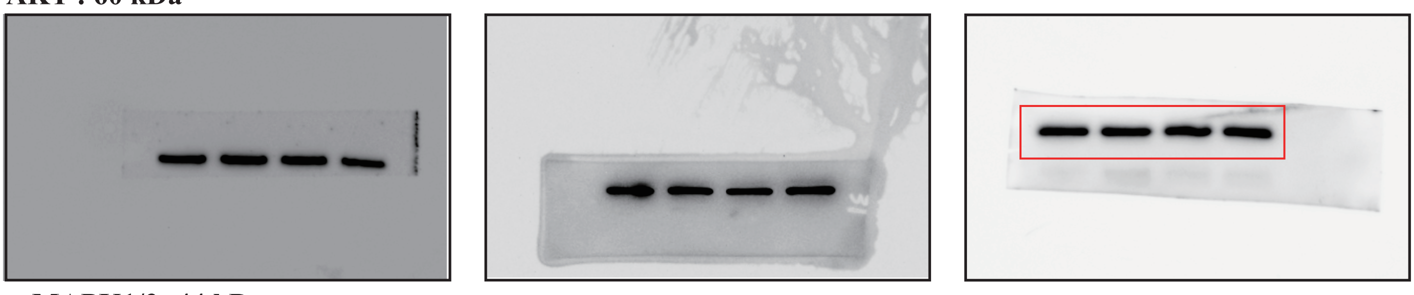

p-MAPK1/3 :44 kDa

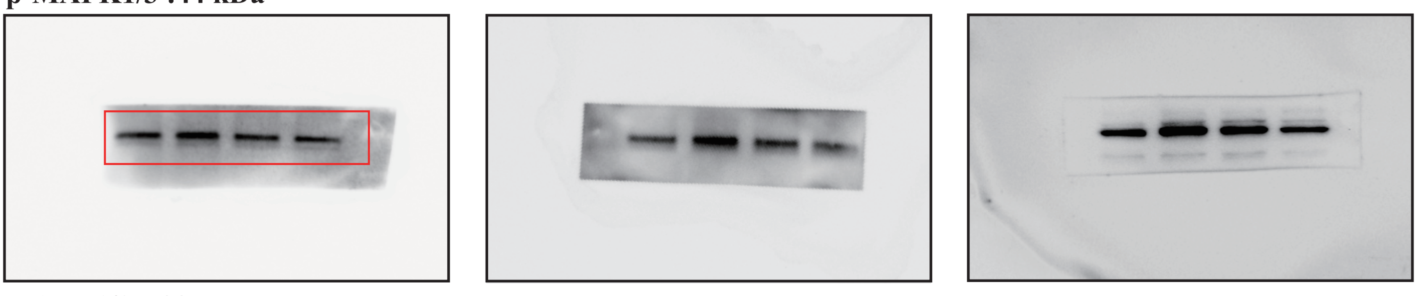

MAPK1/3 : 44 kDa

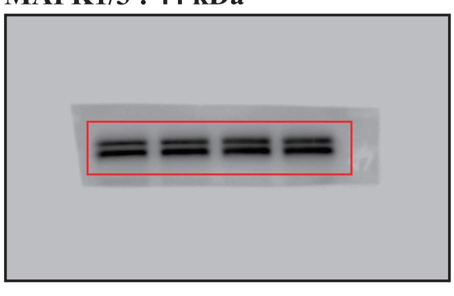

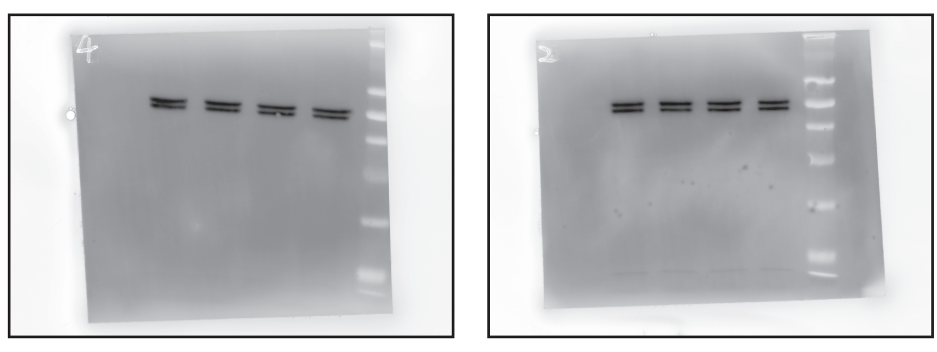

p-NF-κB p65 : 65 kDa

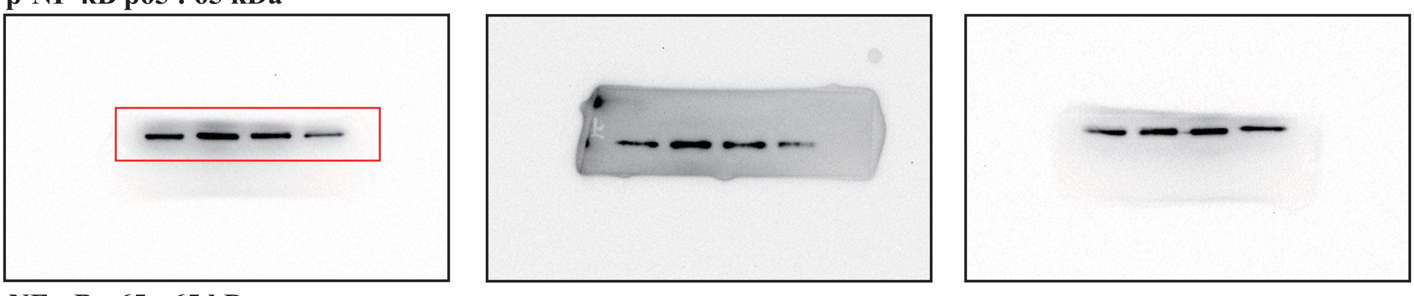

NF-κB p65 : 65 kDa

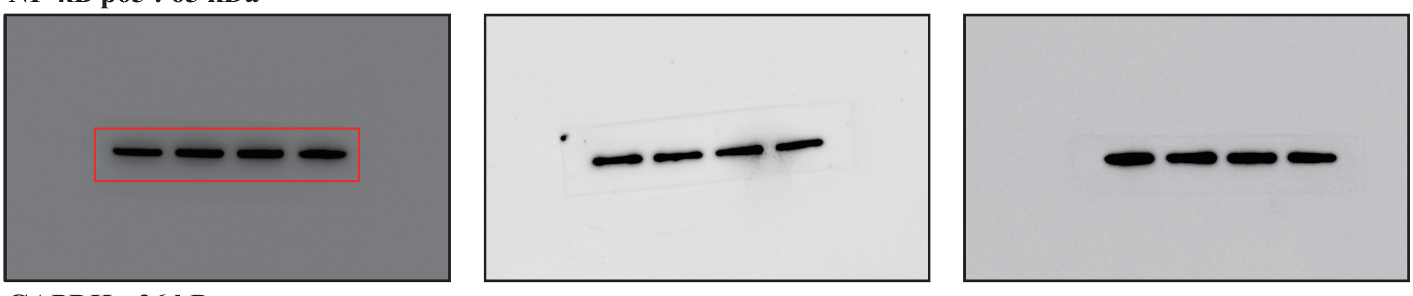

GAPDH : 36 kDa

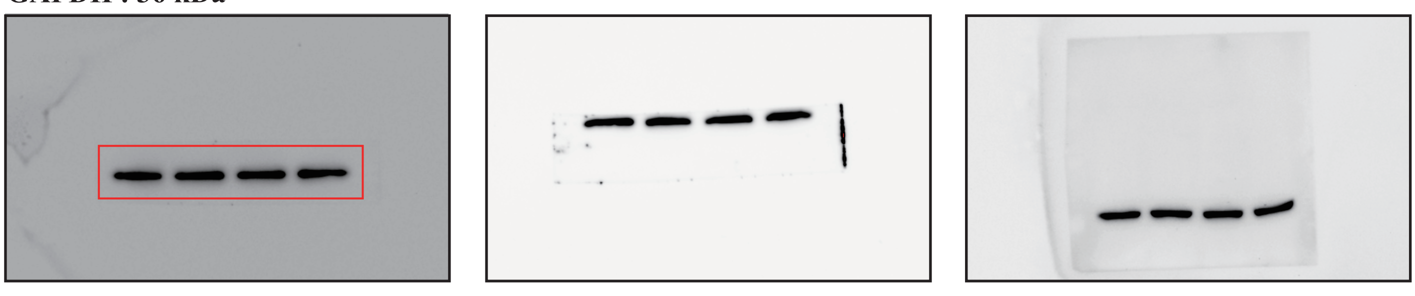

Original images of Supplementary Figure 2

HFF

p-AKT1 : 60 kDa

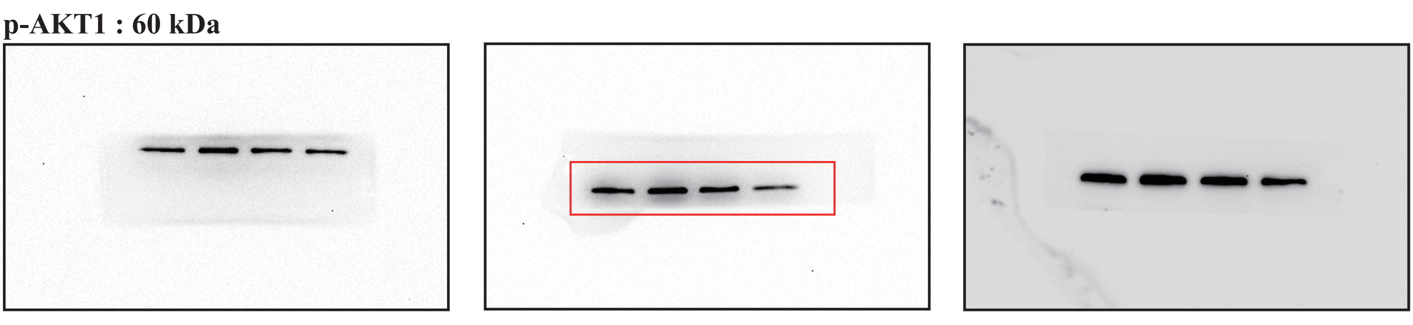

AKT : 60 kDa

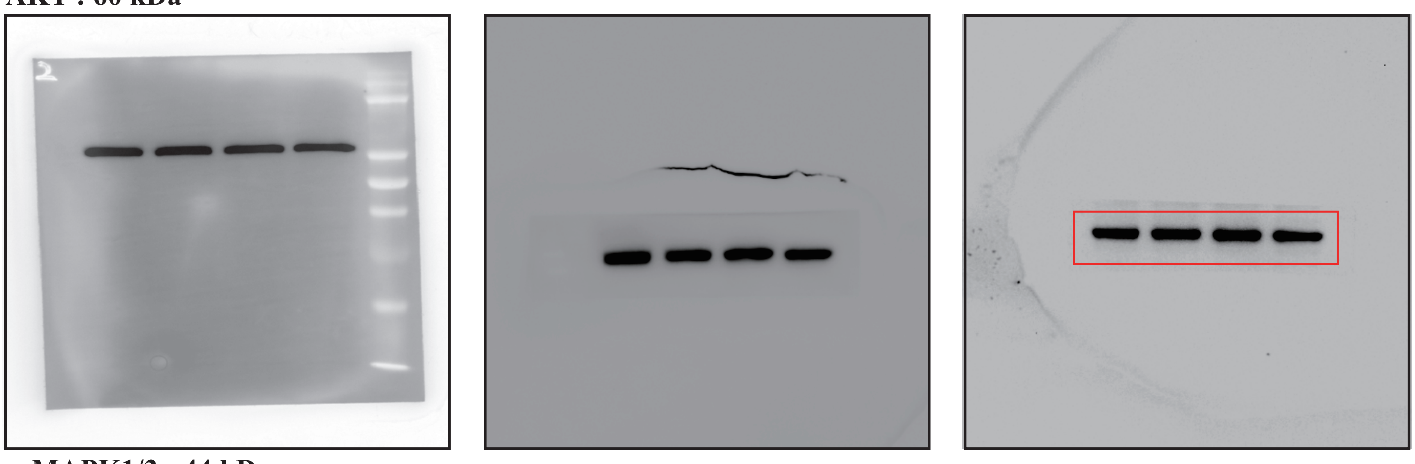

p-MAPK1/3 : 44 kDa

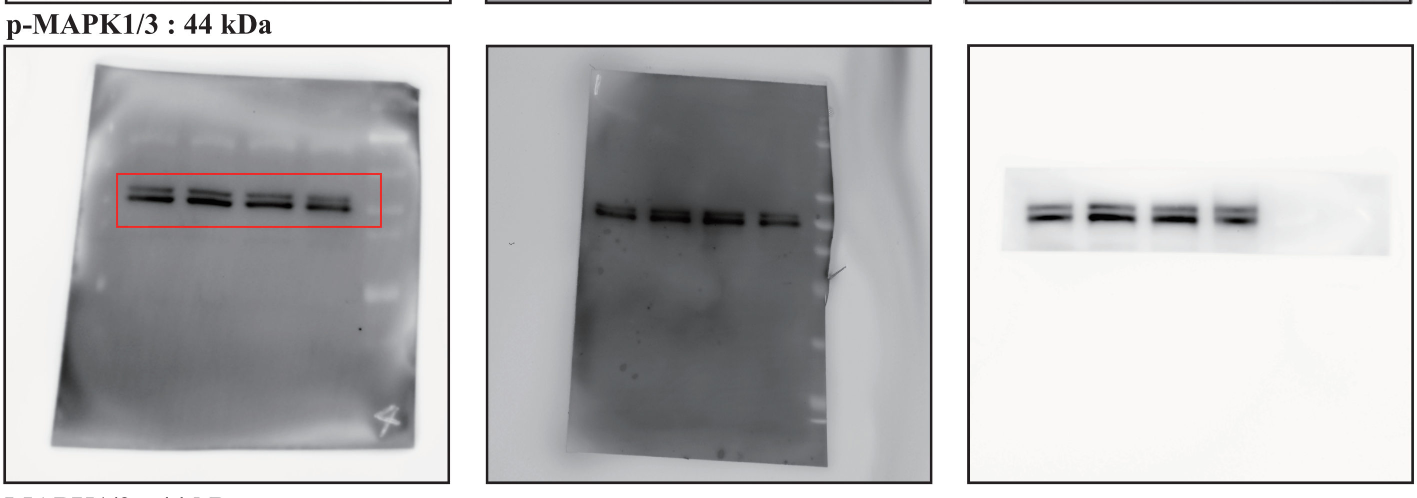

MAPK1/3 : 44 kDa

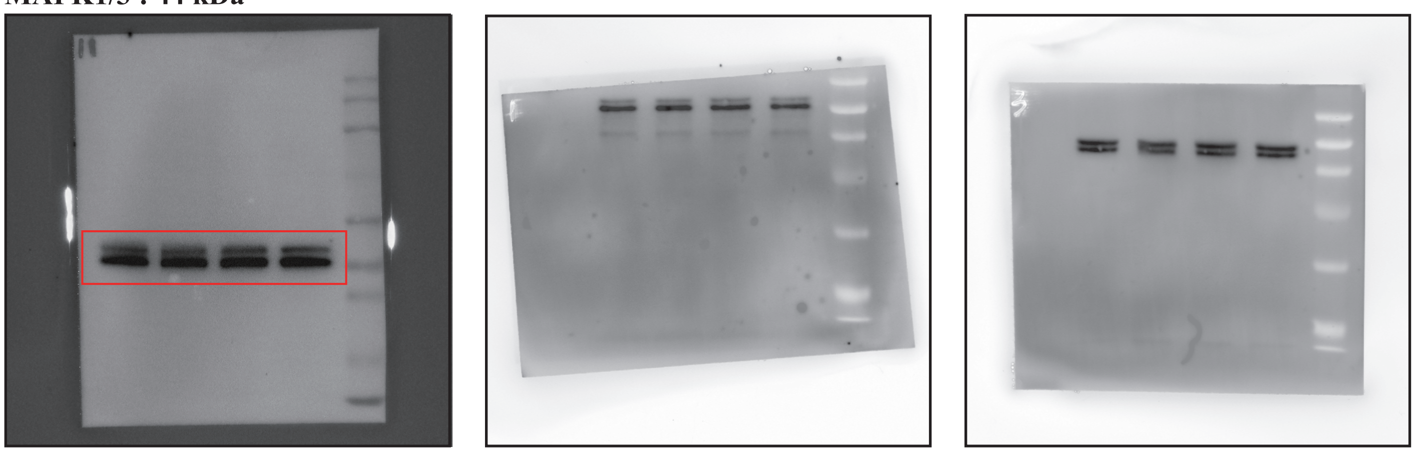

p-NF-κB p65 : 65 kDa

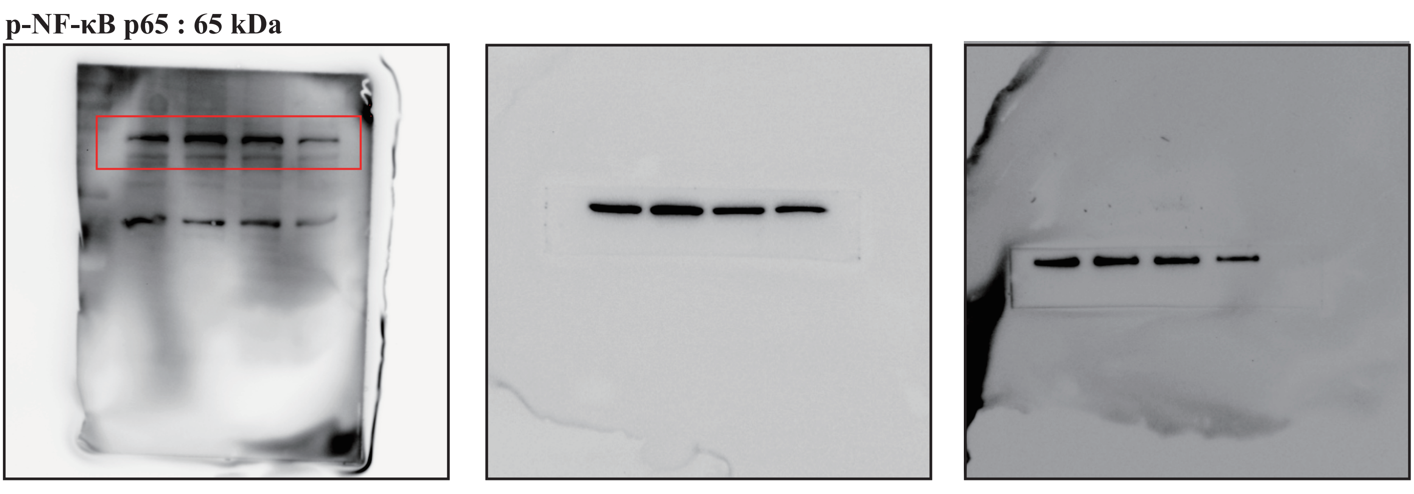

NF-κB p65 : 65 kDa

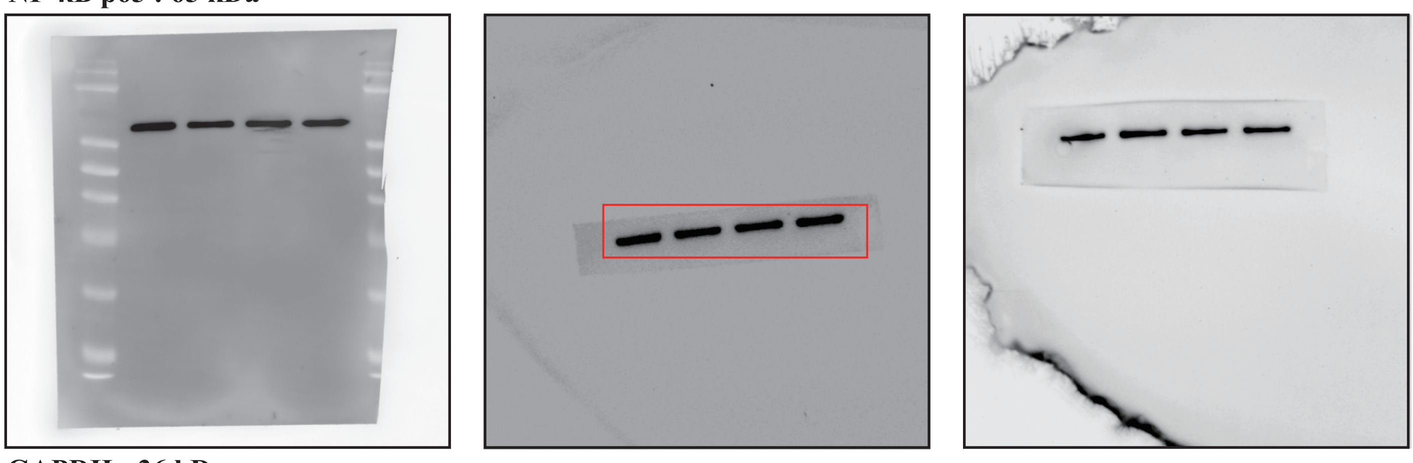

GAPDH : 36 kDa

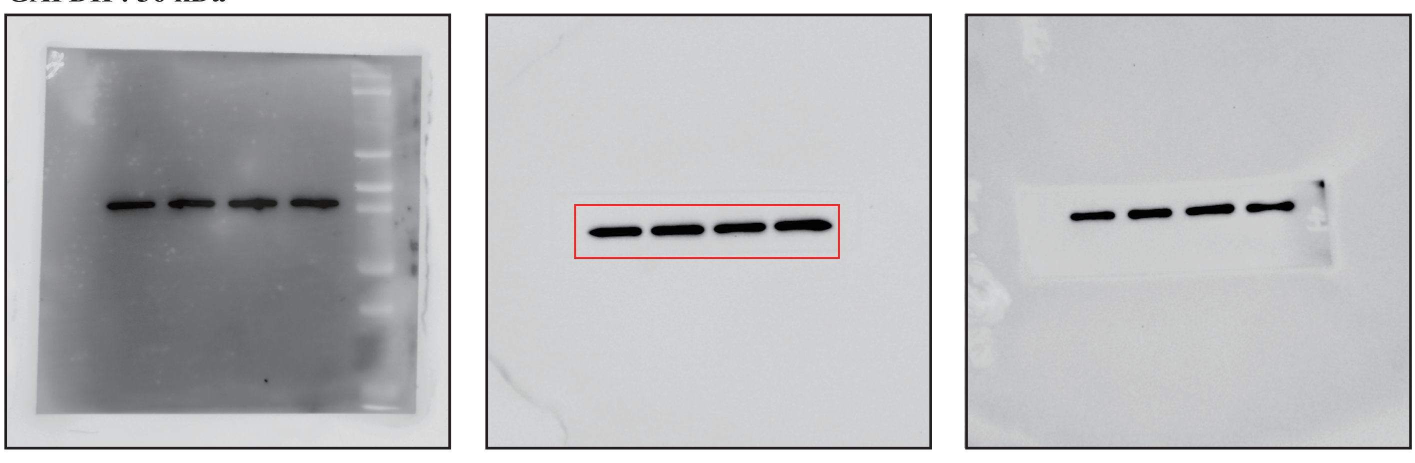

Original images of Supplementary Figure 3

Wound tissue of mice on day 3

p-AKT1 : 60 kDa | **p-MAPK1/3 : 44 kDa** | **p-NF-κB p65 : 65 kDa**

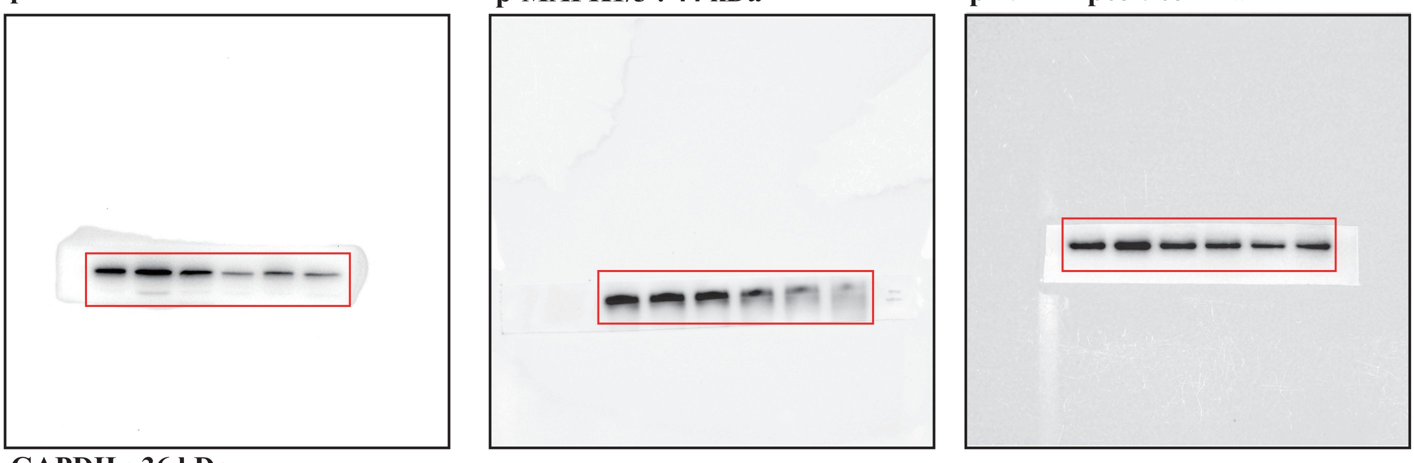

GAPDH : 36 kDa

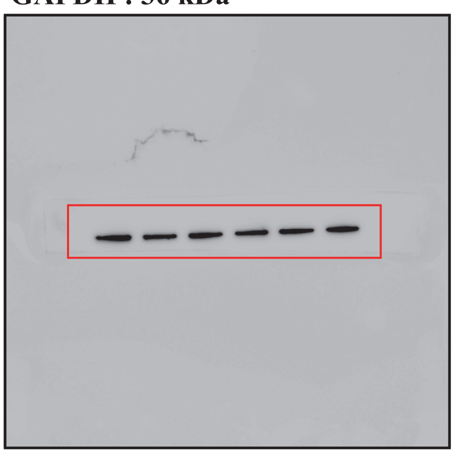

Supplementary Figure 4 the original western blot images. The original blot area used in the main image is indicated by a red box.

Target Gene	Forward primer	Reverse primer
Human IL-6	AGACAGCCACTCACCTCTTCAG	TTCTGCCAGTGCCTCTTTGCTG
Mouse IL-6	TACCACTTCACAAGTCGGAGGC	CTGCAAGTGCATCATCGTTGTTC
Human TNF-A	CTCTTCTGCCTGCTGCACTTTG	ATGGGCTACAGGCTTGTCACTC
Mouse TNF-A	CAGGCGGTGCCTATGTCTC	CGATCACCCCGAAGTTCAGTAG
Human ACTINB	CACCATTGGCAATGAGCGGTTC	AGGTCTTTGCGGATGTCCACGT
Mouse ACTINB	CATTGCTGACAGGATGCAGAAGG	TGCTGGAAGGTGGACAGTGAGG

Supplementary Table 1. The primers for qPCR analysis.

NO	Target	symbol	entrezID
1	Prostaglandin G/H synthase 1	PTGS1	5742
2	Estrogen receptor	ESR1	2099
3	Prostaglandin G/H synthase 2	PTGS2	5743
4	Transcription factor p65	RELA	5970
5	RAC-alpha serine/threonine-protein kinase	AKT1	207
6	Apoptosis regulator Bcl-2	BCL2	596
7	Mitogen-activated protein kinase 3	MAPK3	5595
8	Mitogen-activated protein kinase 1	MAPK1	5594
9	Caspase-3	CASP3	836
10	Fatty acid synthase	FASN	2194
11	Low-density lipoprotein receptor	LDLR	3949
12	Catalase	CAT	847
13	Peroxisome proliferator-activated receptor gamma	PPARG	5468
14	Apolipoprotein B-100	APOB	338
15	3-hydroxy-3-methylglutaryl-coenzyme A reductase	HMGCR	3156
16	Glutathione S-transferase P	GSTP1	2950
17	Peroxisome proliferator-activated receptor alpha	PPARA	5465
18	Sterol regulatory element-binding protein 1	SREBF1	6720
19	Glutathione reductase, mitochondrial	GSR	2936
20	Multidrug resistance-associated protein 1	ABCC1	4363
21	Adiponectin	ADIPOQ	9370
22	Aldo-keto reductase family 1 member C1	AKR1C1	1645
23	C-C motif chemokine 2	CCL2	6347
24	Brain-derived neurotrophic factor	BDNF	627
25	ATP-dependent translocase ABCB1	ABCB1	5243
26	Cytochrome P450 1A2	CYP1A2	1544
27	Cytochrome P450 1B1	CYP1B1	1545
28	Heme oxygenase 1	HMOX1	3162
29	Apoptosis regulator BAX	BAX	581
30	ATP-binding cassette sub-family C member 4	ABCC4	10257
31	Acetylcholinesterase	ACHE	43
32	Actin, aortic smooth muscle	ACTA2	59
33	Alpha-fetoprotein	AFP	174
34	Aryl hydrocarbon receptor	AHR	196
35	Aldo-keto reductase family 1 member C3	AKR1C3	8644
36	Albumin	ALB	213
37	Alkaline phosphatase, tissue-nonspecific isozyme	ALPL	249
38	Apoptotic protease-activating factor 1	APAF1	317
39	Amyloid-beta precursor protein	APP	351
40	Aquaporin-1	AQP1	358
41	Aquaporin-5	AQP5	362
42	Rho GDP-dissociation inhibitor 1	ARHGDIA	396
43	Aryl hydrocarbon receptor nuclear translocator	ARNT	405
44	Bcl-2 homologous antagonist/killer	BAK1	578
45	Cholinesterase	BCHE	590
46	Bcl-2-like protein 1	BCL2L1	598
47	Caspase-7	CASP7	840
48	Caspase-9	CASP9	842
49	G1/S-specific cyclin-D1	CCND1	595
50	G1/S-specific cyclin-E1	CCNE1	898
51	Cyclin-dependent kinase inhibitor 1	CDKN1A	1026
52	Cystic fibrosis transmembrane conductance regulator	CFTR	1080
53	Carnitine O-palmitoyltransferase 1, liver isoform	CPT1A	1374
54	Cathepsin D	CTSD	1509
55	Interleukin-8	CXCL8	3576
56	Cytochrome c	CYCS	54205
57	Aromatase	CYP19A1	1588
58	Cytochrome P450 1A1	CYP1A1	1543
59	Cytochrome P450 2C19	CYP2C19	1557
60	Cytochrome P450 2E1	CYP2E1	1571
61	Cytochrome P450 3A4	CYP3A4	1576
62	Epidermal growth factor receptor	EGFR	1956
63	Estrogen receptor beta	ESR2	2100
64	Glucose-6-phosphate 1-dehydrogenase	G6PD	2539
65	G-protein coupled estrogen receptor 1	GPER1	2852
66	Alanine aminotransferase 1	GPT	2875
67	Glutathione peroxidase 1	GPX1	2876
68	Glutathione peroxidase 2	GPX2	2877
69	Glutathione peroxidase 3	GPX3	2878
70	Interleukin-6	IL6	3569
71	Potassium voltage-gated channel subfamily H member 2	KCNH2	3757
72	Lactoperoxidase	LPO	4025
73	Mitogen-activated protein kinase kinase kinase 5	MAP3K5	4217
74	Mitogen-activated protein kinase 8	MAPK8	5599
75	Mitogen-activated protein kinase 9	MAPK9	5601
76	72 kDa type IV collagenase	MMP2	4313
77	Matrix metalloproteinase-9	MMP9	4318
78	Homeobox protein NANOG	NANOG	79923
79	Nuclear factor erythroid 2-related factor 2	NFE2L2	4780
80	NF-kappa-B inhibitor alpha	NFKBIA	4792
81	Nucleoside diphosphate kinase A	NME1	4830
82	Nitric oxide synthase, inducible	NOS2	4843
83	NAD(P)H dehydrogenase [quinone] 1	NQO1	1728
84	Nuclear receptor subfamily 1 group I member 2	NR1I2	8856
85	Poly [ADP-ribose] polymerase 1	PARP1	142
86	Progesterone receptor	PGR	5241
87	Serum paraoxonase/arylesterase 1	PON1	5444
88	Peroxiredoxin-1	PRDX1	5052
89	Protein kinase C alpha type	PRKCA	5578
90	Protein kinase C beta type	PRKCB	5579
91	Protein kinase C gamma type	PRKCG	5582
92	Prominin-1	PROM1	8842
93	Regulatory-associated protein of mTOR	RPTOR	57521
94	Plasminogen activator inhibitor 1	SERPINE1	5054
95	Sucrase-isomaltase, intestinal	SI	6476
96	Monocarboxylate transporter 1	SLC16A1	6566
97	Solute carrier family 2, facilitated glucose transporter member 1	SLC2A1	6513
98	Zinc finger protein SNAI1	SNAI1	6615
99	Zinc finger protein SNAI2	SNAI2	6591
100	Suppressor of cytokine signaling 3	SOCS3	9021
101	Superoxide dismutase	SOD1	6647
102	Superoxide dismutase	SOD2	6648
103	Transcription factor SOX-2	SOX2	6657
104	Osteopontin	SPP1	6696
105	Signal transducer and activator of transcription 3	STAT3	6774
106	Sulfotransferase 1A3	SULT1A3	6818
107	Transforming growth factor beta-1 proprotein	TGFB1	7040
108	Tumor necrosis factor	TNF	7124
109	Cellular tumor antigen p53	TP53	7157
110	Mitochondrial brown fat uncoupling protein 1	UCP1	7350
111	Vascular cell adhesion protein 1	VCAM1	7412
112	17-beta-hydroxysteroid dehydrogenase type 1	HSD17B1	3292
113	Adenosine receptor A3	ADORA3	140
114	Broad substrate specificity ATP-binding cassette transporter ABCG2	ABCG2	9429
115	Macrophage metalloelastase	MMP12	4321
116	Collagenase 3	MMP13	4322
117	Phospholipase A2, membrane associated	PLA2G2A	5320
118	Group 10 secretory phospholipase A2	PLA2G10	8399
119	Neuronal acetylcholine receptor subunit alpha-7	CHRNA7	1139
120	Kallikrein-1	KLK1	3816
121	Retinoic acid receptor RXR-alpha	RXRA	6256
122	Proto-oncogene tyrosine-protein kinase Src	SRC	6714
123	Mast/stem cell growth factor receptor Kit	KIT	3815
124	Vascular endothelial growth factor receptor 2	KDR	3791
125	Fibroblast growth factor receptor 1	FGFR1	2260
126	Hepatocyte growth factor receptor	MET	4233
127	Carbonic anhydrase 3	CA3	761
128	Carbonic anhydrase 6	CA6	765
129	Cathepsin B	CTSB	1508
130	NADPH oxidase 4	NOX4	50507
131	Aldo-keto reductase family 1 member B1	AKR1B1	231
132	Adenosine receptor A1	ADORA1	134
133	Carbonic anhydrase 9	CA9	768
134	Steroid hormone receptor ERR1	ESRRA	2101
135	Cyclin-dependent kinase 5 activator 1	CDK5R1	8851
136	Insulin-like growth factor 1 receptor	IGF1R	3480
137	Insulin receptor	INSR	3643
138	Dual specificity tyrosine-phosphorylation-regulated kinase 1A	DYRK1A	1859
139	Insulin-like growth factor-binding protein 3	IGFBP3	3486
140	14-3-3 protein gamma	YWHAG	7532
141	NAD-dependent protein deacetylase sirtuin-2	SIRT2	22933
142	Tissue factor	F3	2152
143	Prostaglandin E2 receptor EP1 subtype	PTGER1	5731
144	Prostaglandin E2 receptor EP2 subtype	PTGER2	5732
145	Phosphatidylinositol 4,5-bisphosphate 3-kinase catalytic subunit beta isoform	PIK3CB	5291
146	Phosphatidylinositol 4,5-bisphosphate 3-kinase catalytic subunit alpha isoform	PIK3CA	5290
147	Transitional endoplasmic reticulum ATPase	VCP	7415
148	Placenta growth factor	PGF	5228
149	Vascular endothelial growth factor A	VEGFA	7422
150	Endothelin-1 receptor	EDNRA	1909
151	Serine/threonine-protein kinase/endoribonuclease IRE1	ERN1	2081
152	Stromelysin-1	MMP3	4314
153	Glycogen synthase kinase-3 beta	GSK3B	2932
154	Wee1-like protein kinase	WEE1	7465
155	Tyrosine-protein kinase Lck	LCK	3932
156	Tyrosine-protein kinase SYK	SYK	6850
157	Cyclin-dependent kinase 4	CDK4	1019
158	Aurora kinase A	AURKA	6790
159	Alpha-synuclein	SNCA	6622
160	Polyunsaturated fatty acid lipoxygenase ALOX12	ALOX12	239
161	Hepatocyte nuclear factor 4-alpha	HNF4A	3172
162	Cyclin-dependent kinase 1	CDK1	983
163	Beta-adrenergic receptor kinase 1	GRK2	156

Supplementary Table 2. The 163 intersecting target genes of naringenin treatment for chronic wounds.
